# *Caenorhabditis elegans* and *Drosophila melanogaster* as Model Organisms in Biofactor Research

**DOI:** 10.1002/biof.70127

**Published:** 2026-07-06

**Authors:** Kai Lüersen, Thomas Roeder, Gerald Rimbach

**Affiliations:** ^1^ Department of Food Science Institute of Human Nutrition and Food Science, University of Kiel Kiel Germany; ^2^ Division of Molecular Physiology Institute of Zoology, University of Kiel Kiel Germany; ^3^ ARCN Airway Research Center North of the DZL (German Lung Center) Kiel Germany

**Keywords:** biofactor metabolism, *Caenorhabditis elegans*, chemically defined diets, comparative physiology, *Drosophila melanogaster*

## Abstract

Model organisms play a central role in advancing our understanding of human nutrition and physiology. This narrative review highlights the unique advantages of the invertebrate models *Caenorhabditis elegans* and *Drosophila melanogaster* for studying biofactors—diet‐derived compounds that modulate biological functions. Both models offer powerful genetic toolkits, short life cycles, and cost‐effective maintenance, enabling high‐throughput and mechanistic investigations. Particular emphasis is placed on the availability of chemically defined diets, which allow precise manipulation of nutrient and biofactor composition. These features make *C. elegans* and *D. melanogaster* invaluable systems for dissecting the essentiality, bioavailability, and physiological impact of individual biofactors as illustrated by selected recent studies. Being ethically less problematic substitutes for mammalian models, both invertebrate models offer the possibility of closing the gap that often exists between in vitro and cell culture studies on the one hand and in vivo evaluation in animal experiments in laboratory rodents on the other. By integrating these model systems into experimental research, we can gain critical insights into biofactor‐gene interactions and the molecular mechanisms underlying their health‐promoting properties.

## Graphical abstract

This review presents *Caenorhabditis elegans* and *Drosophila melanogaster* as powerful and ethically sound invertebrate model organisms that can be used to investigate nutritional science questions relating to biofactors. Such nutritional phenotyping studies can bridge the gap between in vitro studies and in vivo research in mammals.

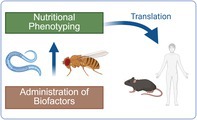

## 1 Introduction

Humans, like all living organisms, depend on an adequate intake of nutrients to meet the demand for fundamental life processes such as development, growth, reproduction, locomotion, a functional immune system, and tissue repair. Macronutrients comprising proteins, carbohydrates and lipids serve as the main sources of energy for the cellular metabolism and as building blocks for body structures [1]. Apart from that, the organism incorporates micronutrients (vitamins and minerals in particular) and a bunch of other diet‐derived molecules, which are supplemented by products of the intestinal microbiota. Collectively, these diet‐derived compounds are referred to as biofactors [2]. In recent years, there has been growing scientific interest in identifying and characterizing biofactors as many of them play essential or modulatory roles in biological systems. In this way, biofactors may be linked, for example, to the maintenance of metabolic homeostasis, prevention of chronic diseases, and promotion of healthy aging. However, elucidating their mechanistic effects, bioavailability, and essentiality in vivo presents a considerable challenge in biofactor research also due to ethical and logistical limitations. In this context, model organisms have become valuable tools, offering controlled, cost‐effective systems for dissecting the complex interactions between diet, metabolism, and genetic background [3]. Among them, the invertebrates *Caenorhabditis elegans* and *Drosophila melanogaster* have emerged as particularly model organisms [4, 5]. In other life science disciplines, research with these two organisms has long made important contributions in uncovering fundamental biological principles [6, 7]. Consequently, there is a wide range of methods and resources for them that are also of interest in the context of experimental nutrition and physiology [8, 9]. For instance, both *C. elegans* and *D. melanogaster* exhibit relatively short lifespans, well‐annotated genomes, and powerful genetic toolkits, which enable high‐throughput and mechanistic studies on a molecular level. The recent development of chemically defined media for both species now allows precise manipulation of dietary inputs, facilitating the study of individual biofactors under controlled experimental conditions [10, 11, 12, 13, 14]. This review critically evaluates the strengths and limitations of *C. elegans* and *D. melanogaster* as model systems in biofactor research, with a focus on their translational relevance, and suitability for mechanistic nutritional investigations.

## 2 The Role of Biofactors in Human Nutrition and Physiology

Biofactors (often synonymously referred to as bioactives or biofunctionals) have been defined as naturally occurring compounds that are either required by the body or can regulate or influence biological functions in a beneficial manner [2]. Hence, biofactors can be essential or non‐essential. In the case of essential biofactors, an under‐supply leads to symptoms of deficiency that cannot usually be compensated for by other nutritional factors. In contrast, non‐essential biofactors are not required for normal body function; however, they support metabolism and/or exert beneficial, health‐promoting effects if the supply of essential nutrients is ensured. Most groups among the biofactors are organic compounds such as vitamins, amino acids, and related biogenic amines, fatty acids, coenzymes, polyphenols, and carotenoids. In addition, there are the inorganic biofactors, such as salts and minerals that are also required for essential and non‐essential metabolic tasks. Since the intestinal microbiota contributes to the supply of nutrients, the portfolio of biofactors can also be indirectly modulated or expanded by influencing the microbiota through the intake of probiotics and prebiotics [15, 16].

Certain food categories are particularly suitable as sources of the various biofactors. Fruits and vegetables, for example, are usually rich in vitamins, polyphenols, and carotenoids. Whole grains and legumes can provide fiber, minerals, and prebiotics. Dairy and fermented foods contain high levels of probiotics, calcium, and B vitamins. Nuts, seeds, and fish are good sources of protein, omega‐3 fatty acids, and minerals. Herbs and spices are known for their high content of specific polyphenols [17].

Due to their great diversity, it is not surprising that biofactors influence virtually all aspects of life including processes such as strengthening energy metabolism, improving the immune system, protecting against and detoxifying reactive oxygen species (ROS), positively influencing brain function and cognition, stabilizing mood, maintaining gut health and aiding digestion, in addition to supporting heart, skin, and bone health [2]. For those biofactors that have so far been considered essential, it is generally possible to assign one or more specific tasks in metabolism. Compared to the totality of all biofactors, however, they represent only a minority, and it remains a goal in nutrition research to investigate whether additional biofactors extend this list of essential compounds. Among the trace elements and ultra‐trace elements, for example, there are some members such as vanadium, nickel, lithium, or boron that are considered to be potential essential nutritional factors, although there is no clear proof of this to date [18].

In contrast, secondary metabolites of plant or fungal origin, probably the largest group of biofactors, are most likely non‐essential dietary factors, which are nevertheless generally considered to have a positive effect on the organism. It is currently assumed that of the 100,000 known secondary metabolites [19], around 5000–10,000 are part of the human diet [20, 21]. Their spectrum can be extended by teas, infusions and extracts of herbs and medicinal plants. The secondary metabolites include such diverse compounds as polyphenols, carotenoids, mono‐ and diterpenes, triterpenoids, phytosterols, phytoestrogens, glucosinolates, betalains, sulphides, amines, saponins, protease inhibitors, lectins, chlorophyll, and phytic acid. Knowledge of these biofactors has already improved significantly in recent years, thanks to human epidemiological studies and experimental intervention studies in animal models and humans. Many secondary metabolites have been reported to exhibit beneficial effects, often in the context of age‐related diseases such as cardiovascular diseases, cancer, diabetes, neurodegeneration, and metabolic liver diseases [2, 20, 22]. Consequently, such biofactors are attributed to slow down the aging process or at least support healthy aging. In addition, some secondary metabolites influence the digestion and absorption of nutrients either by stimulating or slowing down these processes. Especially the inhibition of carbohydrate and fat breakdown and absorption in the intestine by biofactors can prove to be beneficial against the background of the spreading consumption of the so‐called Western‐type diet [23, 24].

On the other hand, it must be taken into account that several classes of phytochemicals, such as lectins, phytic acid, tannins, cyanogenic glycosides, and saponins, were found to act as anti‐nutrients or even toxins [25]. Moreover, it has been shown that secondary plant metabolites often suffer from the shortcoming of poor in vivo bioavailability due to instability, low solubility, and/or permeability [26]. Accordingly, there are still many unanswered questions in this area. Are these biofactors effective when they are given in an isolated form? Do they show opposing, additive, or even synergistic effects when given in combination? What are their side effects, especially when given at high doses [27] or when their bioavailability is improved, for example, by nanotechnological means [28]? Moreover, for most of the secondary metabolites, the mechanism of action is only barely understood.

In summary, a proper supply of essential nutrients supports the body's functions and prevents metabolic stress. In addition, many biofactors in the diet are vital components that go beyond basic nutrients to optimize health and well‐being. Accordingly, it is assumed that a balanced diet with a wide variety of bioactive factors can help prevent chronic diseases and promote overall longevity.

## 3 The Concept of Model Organisms

In the course of the history of life sciences, including nutritional science, researchers have agreed on a few organisms that have a model character in that they are used by many research groups to investigate fundamental biological processes. These model organisms help generate hypotheses that are later tested in more complex systems, while also serving to validate findings from in vitro and cell culture studies in living organisms [29, 30]. A key requirement for a model organism is that it is easy to cultivate and handle experimentally. Widespread use across research groups leads to a growing body of knowledge, established methodologies, and shared infrastructure such as databases and stock centers. This collective focus strengthens their status as models.

In his Nobel Prize speech, Sydney Brenner [31] summarized the model organism question as follows: “…choosing the right organism for one's research is as important as finding the right problems to work on…”. Hence, when selecting a model organism, several key factors must be taken into account. (i) Simpler organisms are often preferred for addressing specific research questions. (ii) The organism should possess relevant traits that are easy to analyze. (iii) Findings must be transferable to more complex organisms, often humans, making phylogenetic position important. (iv) The limits of model organisms must be acknowledged, as the applicability of results—especially to humans—requires careful, case‐by‐case evaluation.

### 3.1 Model Organisms in Nutritional Research

Experimental nutritional research aims to decipher the influence of feeding behavior, dietary patterns, and biofactors on human metabolism, body composition, and health [1]. It is obvious that for ethical, financial, and practical reasons, it is often neither sensible nor feasible to investigate nutritional questions on topics such as nutrient requirements, essentiality of nutrients, or toxicity of biofactors directly in humans. Therefore, the use of animal models in experimental nutritional research has a long tradition, leading, for example, to the discovery and identification of vitamins, essential minerals, or hormones and the elucidation of their physiological significance [3].

These early studies from the 19th and early 20th century were mainly carried out in mammalian species such as dogs, cows, pigs, or guinea pigs but also chickens [3, 32]. However, later laboratory rodents such as rats and mice as well as rabbits became the most commonly used models in experimental nutritional research [32]. Being mammals, these animal species are closely related to humans in evolutionary terms, sharing ~95% of human genes. This is also reflected in similarities in their anatomy (gastrointestinal tract, liver, closed circulatory system, lungs, heart, kidneys, and other organs), immune system (innate and adaptive immune system), and metabolism. A good example of its suitability to map human‐relevant aspects of metabolism is shown by the fact that the discovery of the obese *ob/ob* mouse in the Jackson Laboratories in 1949, the *db/db* mouse in 1966 [33] or the Zucker fatty rat in 1961 [34] not only provided genetic metabolic disease models but also led to the identification of the genes for leptin and its corresponding leptin receptor, which are functionally well conserved in humans. In addition, the development of techniques for the genetic manipulation, particularly in mice, has generated an impressive range of genetic (disease) models that can be studied in a nutritional context.

However, maintaining and breeding large numbers of laboratory rodents is relatively expensive, labor‐intensive and requires a lot of space. In addition, safety and ethical aspects have become increasingly important in recent years, limiting the use of mammalian model animals. Legal requirements led to mandatory protocols for animal handling concerning minimizing pain, suffering/distress and killing [35]. The “three R” principles “Reduction” (reducing the number of animals required to obtain information to a certain extent and with a certain accuracy), “Refinement” (refining procedures to minimize the frequency or severity of animal suffering), and “Replacement” (replacing conscious, living vertebrates with insentient alternatives) (often extended by a fourth R referring to “responsibility”) established already in 1959 [36] serve as a guideline according to which alternatives for the use of vertebrate laboratory animals must be sought and tested for their practicability and transferability. In order to be used widely as a model in nutrition research, an organism should fulfill certain requirements that enable a simple investigation of the complex interactions between nutrition, metabolism, genetics and health (Box 1).

BOX 1 **Criteria for a versatile model organism in nutritional science.**

A versatile model organism for nutritional research should enable the investigation of the complex interaction between nutrition, metabolism, genetics and health. In addition, to ensure that findings are relevant and translatable to the human situation, such an organism should meet several important criteria, which are listed below along with other key requirements:

- Physiological and metabolic similarity to humans
- Short life cycle and generation time
- Low cost and ethical accessibility
- Controlled environmental and dietary conditions
- Dietary flexibility allowing different nutritional scenarios.
- Genetic tractability and manipulability
- Established omics tools and genome databases.
- Presence of suitable human disease models.

### 3.2 The Worm and the Fly: Key Invertebrate Model Organisms

In current life sciences, the fruit fly *D. melanogaster* and the nematode *C. elegans* are the most prominent invertebrate model organisms (Table 1). In the early 20th century, Thomas Hunt Morgan and his colleagues and later his disciple Herman J. Muller found that *D. melanogaster* was an excellent organism for studying basic genetics such as chromosome linkage, crossing over, and mutagenesis [37]. In the 1960s, *C. elegans* was selected by Sydney Brenner and his co‐workers to address the question of how genes can govern the complex structures of higher organisms [38]. Hence, both species were initially introduced as genetic models before their applications spread to almost all disciplines in modern life sciences [6, 7]. Given this background, it is not surprising that they were the first multicellular organisms whose genomes were fully decoded [39, 40], which in turn opened the door to the development of various omics approaches.

**TABLE 1:** Comparison of the invertebrate models *C. elegans* and *D. melanogaster* .

Feature/criterion	*Drosophila melanogaster*	*Caenorhabditis elegans*
Taxonomy	Insect (Arthropoda)	Nematode (Nematoda)
Body plan	Segmented, bilateral, three body sections: head, thorax, abdomen	Unsegmented, bilateral, simple tube‐like body
Adult body size	~2–3 mm	~1 mm
Genome size	~165 Mb; ~14,000 genes	~100 Mb; ~20,000 genes
Sequenced genome	Yes (2000)	Yes (1998, first multicellular genome sequenced)
Lifespan	~50–80 days at 25°C	~18–20 days at 20°C
Generation time	~10–12 days at 25°C	~3–4 days at 20°C
Reproduction	Sexual with males and females	Self‐fertilizing hermaphrodites, low incidence of males
Number of offspring	> 300 per female	~300 progeny per hermaphrodite
Developmental stages	Egg → Larva (3 instars) → Pupa → Adult	Embryo → L1–L4 larval stages (alternate dauer larvae) → Adult
Ease of genetic manipulation	High (GAL4/UAS, CRISPR, RNAi, transgenics)	High (RNAi by feeding, CRISPR, transgenics)
Cell number (adult)	~100,000 cells	959 somatic cells in hermaphrodite (eutely)
Neurons	~100,000 neurons	302 neurons (fully mapped connectome)
Behavioral complexity	High (learning, memory, circadian rhythm, courtship)	Moderate (locomotion, chemotaxis, egg‐laying)
Tissue complexity	Complex organs (brain, heart, gut, muscles, eyes, etc.)	Simple tissues (no complex organs or circulatory system)
Imaging and cell tracking	Moderate (especially in early development)	Excellent (transparent body, entire cell lineage known)
Disease modeling	Neurological, metabolic, cancer, aging, immunity	Aging, neurodegeneration, apoptosis, stress response
Ethical considerations	Minimal	Minimal
Costs and space requirements	Low	Very low
Use in drug screening	Moderate to high	High (especially in high‐throughput formats)

The establishment of large research communities has been accompanied by the built‐up of publicly accessible online resources such as the databases Flybase (https://flybase.org) and Wormbase (https://wormbase.org) as well as the foundation of various stock centers Bloomington *Drosophila* Stock Center (BDSC; https://bdsc.indiana.edu/), Kyoto *Drosophila* Stock Center (KDSC; https://kyotofly.kit.jp/cgi‐bin/stocks/index.cgi), Vienna *Drosophila* Resource Center (VDRC; https://shop.vbc.ac.at/vdrc_store), and Korean *Drosophila* Resource Center (http://kdrc.kr/index.php) for *D. melanogaster* and *Caenorhabditis* Genetic Center (CGC; https://cgc.umn.edu) for *C. elegans* that provide mutant strains, transgenes, and other genetic resources. These large collections of mutants and transgenes are of value for deciphering genotype–phenotype relationships in biological processes and for establishing human disease models [8, 9].

Working with both *C. elegans* and *D. melanogaster* is more cost‐effective, less space‐consuming, more ethical, and less restricted by animal law compared to conventional model organisms (e.g., rodents). Additionally, their short lifespans and rapid reproductive cycles enable rapid experimentation and data generation with a large number of individuals. The strength of these two models is also demonstrated by the fact that research with them has so far led to nine Nobel Prizes (four for *C. elegans* research in 2002, 2006, 2008 and 2024 as well as in six for *D. melanogaster* research in 1933, 1946, 1995, 2004, 2011, 2017).

### 3.3 The *C. elegans* Model

*C. elegans* is a small, free‐living nematode primarily found in temperate regions around the world. The worm is about 1 mm long and, at an ambient temperature of 20°C, develops quickly throughout its life cycle from egg to adult worm through four larval stages within 3 days. Under adverse environmental conditions such as food deprivation, the first larval stage can develop into an alternate dauer larva that allows for long‐term survival.

The adult hermaphrodites have a lifespan of 2–3 weeks and usually self‐fertilize, producing up to 300 eggs. Males are present at very low abundances in populations and can inseminate hermaphrodites, which results in higher egg production with up to 1000 progenies [6]. *C. elegans* has a strictly determined development with an invariant cell lineage leading to a constant number of somatic cells (959 for hermaphrodites, 1033 for males), a phenomenon known as eutely. The nematodes are transparent throughout their whole lifecycle [6], which allows detailed live imaging studies of cellular processes, including changes induced by nutrition. The worm has different tissues including muscles, a nervous system, an intestine, an excretory organ, and reproductive systems. *C. elegans* lacks a liver‐like organ and an adipose tissue, which, in other animals, is not only an important energy storage organ, but also has endocrine and immunological functions. Instead, the intestine of *C. elegans* takes over the task of fat storage and also liver functions.

The genome of the worm, which was fully sequenced in 1998, consists of 6 chromosomes encoding for approximately 20,500 genes, with 35% of the genes having homologues in humans [39, 41]. *C. elegans* is a versatile genetic model that can be applied for reverse and forward genetics, including mutagenesis, RNA interference, CRISPR/Cas, and transgenic approaches [9]. Moreover, owing to its small body size and ease of handling, *C. elegans* is one of the few multicellular organisms that allows in vivo high‐throughput screening approaches [42, 43]. *C. elegans* cultures can be synchronized in the egg stage by treating adult worms with a bleach solution [6]. A major advantage of the *C. elegans* model is that the worms can be frozen and stored indefinitely in liquid nitrogen [38].

#### 3.3.1 *C. elegans* : Structure of the Gastrointestinal Tract and Feeding Behavior in Nature

**FIGURE 1:**
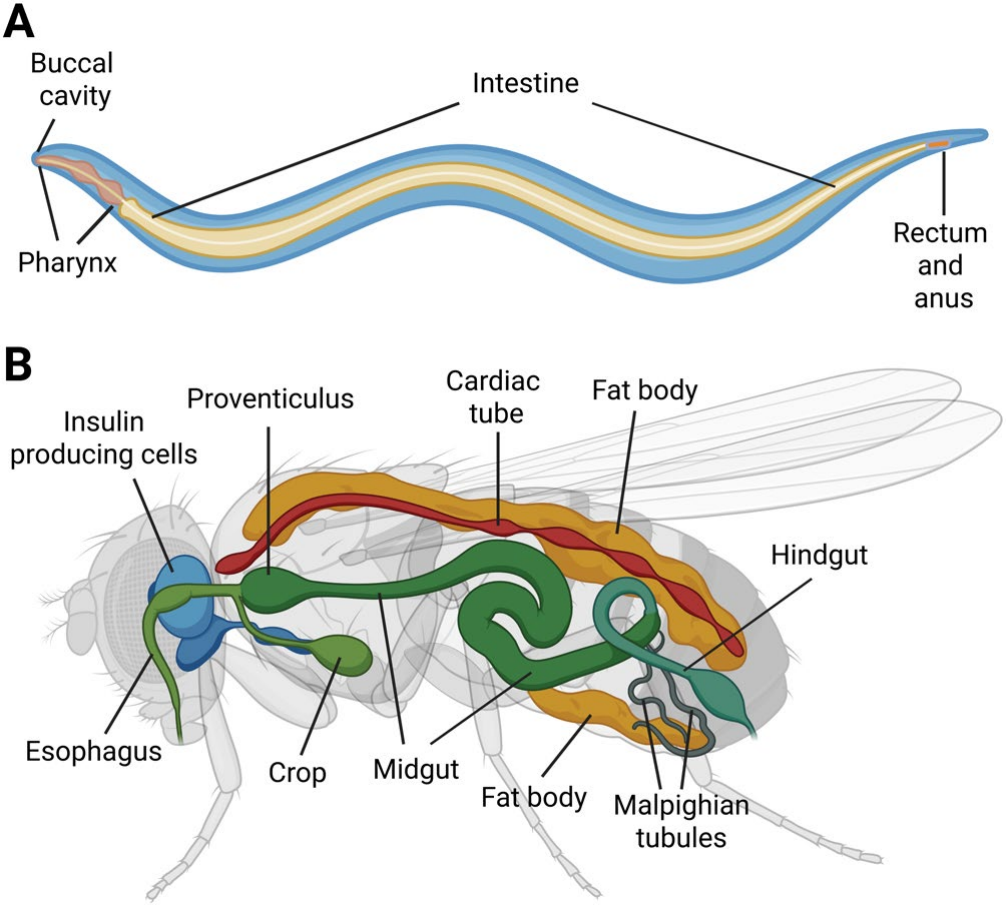
The anatomy of (A) *C. elegans* and (B) *D. melanogaster* with a focus on the alimentary systems. Created in BioRender. Rimbach, G. (2026) https://BioRender.com/nh1ihqx.

In nature, *C. elegans* is found in humid habitats of decaying plant material such as rotting stems, fruits, and flowers. From a nutritional perspective, *C. elegans* is a bacterivore, but can also use particular yeast species as a food source [44, 45]. With the help of its muscular pharynx, which acts as a sucking pump and grinder, *C. elegans* ingests and prepares its food for further digestion within the intestinal lumen. Nutrients are then taken up by the 20 intestinal cells that form the simple gastrointestinal tract (Figure 1A). In addition, field studies have revealed that *C. elegans* possesses a diverse native gut microbiome in natural habitats [46]. Hence, the bacterial food and the compounds delivered by the microbiota community represent the primary source of macro‐ and micronutrients [45].

#### 3.3.2 *C. elegans* Standard Diets in the Laboratory

In the laboratory, *C. elegans* strains are routinely fed with a lawn of the uracil‐auxotroph *Escherichia coli* strain OP50 grown on nematode growth medium (NGM) agar (0.2% peptone, 1.6% agar, 0.24% NaCl, 1 mM CaCl_2_, 1 mM MgSO_4_, 25 mM K‐PO_4_‐buffer, 5 mg/L cholesterol) plates. Alternatively, the nematodes can be maintained in liquid culture consisting of liquid NGM inoculated with the *E. coli* strain HB101 [47]. By increasing the carbohydrate concentration in the NGM agar, high‐sugar diets have been established that could increase fat accumulation in *C. elegans* [48, 49].

It is evident that the bacterial food source can complicate and confound nutritional studies in many ways, as the prokaryote has a metabolism of its own and contains relatively fixed amounts of the macronutrients. Therefore, approaches to design more defined *C. elegans* diets aimed to exclude the food bacteria. A complex axenic media containing 3% soy peptone, 3% yeast extract and 0.5 mg/mL of hemoglobin supports *C. elegans* growth [50, 51]. However, several lines of evidence suggest that worms fed with this medium face a severe food restriction, as their development is slowed, their fecundity is reduced and their lifespan is prolonged. Possible causes include a lack of critical nutrients that are normally provided by food bacteria or an unfavorable nutrient geometry. A generation time comparable to the standard cultivation on *E. coli* OP50 NGM plates was achieved with a different axenic medium [52]. This *C. elegans* Habituation and Reproduction (CeHR) medium is mostly chemically defined food but requires skim milk as an essential additive and thus is a semi‐defined medium.

### 3.4 The *D. melanogaster* Model

The fruit fly *D. melanogaster* is a globally distributed insect [53]. The adult female measures around 2.5 mm in length, while the males are somewhat smaller. Its holometabolic life cycle, which in the laboratory takes about 10 days at 25°C, comprises the four life stages: egg, larva, pupa, and imago (or adult). After mating, females lay up to 300 eggs (egg size is about 0.5 mm), from which first instar larvae hatch, which develop through two further larval stages to the pupa. Metamorphosis then takes place in the pupa, from which the imago emerges. *D. melanogaster* cultures can be synchronized starting from the eggs, and the adult flies can be kept separately by sex or as a mixed population. Depending on the natural isolate, adult flies have different, often sex‐specific life expectancies, which typically range from 50 to 80 days. Thus, similar to *C. elegans* , *Drosophila* allows for both short‐ and long‐term studies within a reasonable timeframe, making it ideal for aging, longevity, and transgenerational research [4, 8, 53].

The segmented adult body is divided into three parts: head, thorax, and abdomen. The fruit fly contains many tissues that have a remarkable functional similarity to human organs. These include a central nervous system, gastrointestinal system, musculature, adipose tissue, the liver‐equivalent fat body, a cardiac tube (heart‐equivalent), a respiratory system (trachea), and the kidney‐equivalent Malpighian tubules [4, 8, 53].

The diploid genome is organized in three pairs of autosomes and one pair of sex chromosomes. The analysis of the genome, after its sequencing was completed in the year 2000, revealed 13,000 genes, with 60% of them having homologues in humans [40]. Similar to *C. elegans* , *D. melanogaster* is a compelling genetic model for both reverse and forward genetics, including mutagenesis, RNA interference, CRISPR/Cas, and transgenic approaches, at the organismal or tissue‐specific level [8]. Powerful binary expression systems, such as the UAS‐Gal4 or the recently introduced Q expression system, play a crucial role here [54, 55]. These systems rely on so‐called driver lines that express a transcription activator protein (for instance, the yeast transcription activator protein Gal4) under the control of a specific *D. melanogaster* promoter, along with corresponding reporter/effector lines that contain genes of interest governed by the related specific cis‐acting enhancer binding sequence. In the case of Gal4, for example, this is the upstream activation sequence (UAS).

Finally, in contrast to *C. elegans* , cell cultures and various methods of genetic manipulation, such as cell transfection, have been established for the fruit fly, which can be used, for example, to facilitate the elucidation of molecular mechanisms [56]. Currently, approximately 250 *Drosophila* cell lines are available through the BDSC (https://dgrc.bio.indiana.edu/cells/Catalog). Only recently, *D. melanogaster* intestinal cell lines have been established using a transgenic approach, in which the oncogene Ras^V12^ was specifically expressed in the intestine of late embryos [57].

#### 3.4.1 *D. melanogaster* : Structure of the Gastrointestinal Tract and Feeding Behavior in Nature

In nature, *D. melanogaster* feeds on fruit and rotting, fermenting organic material. These habitats are rich in yeast and other microbes, which are primarily consumed as food by larvae or adult flies; however, the liquid or pulpy parts of the sugary fruit itself are also ingested [53, 58]. In contrast to *C. elegans* , adult fruit flies can actively search for food sources. The food is consumed by a pumping mechanism driven by the muscular parts of the fly's proboscis (mouthparts), whereby swallowing through the pharynx is triggered in particular by the suction and compression of the cibarium [59]. In contrast to the simple structure of the *C. elegans* intestinal tract, the gut tract of the fruit fly is a much more complex tissue (Figure 1B), as it consists of several cell types and can be divided into several functional sections [60]. In adult fruit flies, a distinction is made between the (i) foregut, which can be subdivided into esophagus, crop, and cardia (or proventriculus), (ii) the midgut, which consists of six major anatomical regions with 10–14 morphologically and molecularly distinguishable subregions, where most of the digestion and absorption takes place, and (iii) the hindgut with pylorus, ileum, and rectum, where water/ion exchange may occur.

The intestinal tube is lined by an epithelial monolayer comprising various cell types, including intestinal stem cells, progenitor cells, enterocytes, and enteroendocrine cells. The intestinal stem cells give rise to two progenitor cells: enteroblasts and enteroendocrine progenitor cells. These progenitor cells develop into absorptive enterocytes and secretory enteroendocrine cells, respectively. Hence, intestinal stem cells are responsible for tissue regeneration [61]. The enterocytes produce and secrete digestive enzymes and are involved in absorbing and transporting nutrients from the lumen into the hemolymph. Based on bioinformatic analyses of genome and transcript data, it is assumed that the intestine of the fruit fly can produce around 350 digestive enzymes mainly for the breakdown of proteins/peptides, carbohydrates, and lipids [60].

The secretory enteroendocrine cells (EECs) function as sensors of luminal content and, in response to that, produce and release hormones and neurotransmitters regulating numerous physiological processes, including appetite, food ingestion, food digestion, gut motility, metabolism, and immune responses [62]. The intestinal epithelium secretes mucus into the lumen of the intestine and a chitinous layer, the peritrophic matrix, produced by the cardia in the posterior part of the foregut [60]. The intestinal tract is associated with visceral muscles, is innervated by neurons, and is supplied with oxygen by the tracheal system. In addition, the excretory Malpighian tubes join at the connection between the midgut and hindgut [60].

Similar to *C. elegans* , the intestine of *D. melanogaster* is colonized by a microbiota [63, 64]. However, when compared to mammals, which usually host more than 500 species in their gastrointestinal tract, the gut microbiome of fruit flies is much simpler. Laboratory *D. melanogaster* strains usually harbor about 10 different species, predominantly from the phyla *Proteobacteria* and *Firmicutes*. The composition of the microbiota influences numerous processes in the fruit fly, such as immune status, stress resistance, reproduction, development, and life expectancy. From a nutritional point of view, it is interesting to note that the microbes can support the breakdown of nutrients from the ingested food and deliver metabolic products to their host. On the other hand, the quality and quantity of the fruit fly diet shapes the composition and abundance of the bacterial community in the gut. Of note, *Drosophila* can be cultured under microorganism‐free (axenic) conditions or with defined microbial communities (gnotobiotic) [65].

#### 3.4.2 *D. melanogaster* Standard Diets in the Laboratory

In contrast to the bacteria feeder *C. elegans* , no microbes need to be added to the diet of *D. melanogaster*, as the fruit fly, accepts a wide range of axenic complex feeds when maintained under laboratory conditions [66, 67]. Most diets are formulated based on yeast, corn meal, sucrose, and agar, whereby the nutrient composition can vary considerably among the recipes. In addition, other ingredients such as glucose, barley, soy, peptone, and bananas are sometimes used, which together with batch‐to‐batch variations in the natural ingredients lead to an even higher heterogeneity in macronutrients (proteins, carbohydrates, and lipids) and (essential) micronutrients (vitamins and minerals). This frequently complicates or even compromises the comparability of nutritional studies on *D. melanogaster* [67].

Nevertheless, in contrast to the situation in *C. elegans* , the composition of these complex *Drosophila* diets can be modified relatively easily with respect to the macronutrients sugar, fat, and protein. Lee et al. [68] and Morimoto et al. [69] used a diet that consists solely of sucrose and yeast extract to vary the dietary carbohydrate to protein ratios and study the impact of the geometry of both macronutrients on the fitness parameters reproduction, life expectancy, and male attractiveness. However, it should be kept in mind that the yeast extract is not only the protein source of the feed but also provides almost all micronutrients including vitamins and minerals. High‐sugar diets, in particular, have been successfully used to induce metabolic disorder states in the fruit fly, which phenocopy the human situation to a high extent [70, 71]. But again, there is no standardized high‐sugar diet. Rather, different sugar concentrations and different types of sugar have been used, some of which have been demonstrated to exert specific effects on the fly [72, 73, 74]. Similarly, high‐fat diets were generated for *Drosophila* feeding experiments by adding different fats, such as lard or coconut oil; however, this was often done without considering the varying fatty acid composition and/or relevant variations that can occur between different batches of lard and coconut oil [75]. Finally, complex diets based on hydrolysed yeast extracts and sucrose or glucose have proved very useful for identifying the mechanisms by which dietary restriction, applied by food dilution, extends lifespan [76].

### 3.5 Chemically Defined Media and Essential Biofactors for *C. elegans* and *D. melanogaster*

It is in the nature of the subject that the composition and modifiability of the diet play a central role in experimental nutrition research [1]. Therefore, a diet that is fully defined in its composition and covers all nutritional requirements is of great value. This must also be taken into account when selecting the model organism if it is to be used to elucidate the function of biofactors (especially since a defined diet is not practicable in humans). Such defined media have been developed for both *C. elegans* and *D. melanogaster* .

The *C. elegans* minimal medium (CeMM) consists of 54 ingredients [14] and can be provided in solid or liquid form [13]. It is noteworthy that if fed as a liquid, the medium must contain particular matter. Otherwise, the particle feeder *C. elegans* , which filters the ingested food by actively expelling liquid, is unable to absorb sufficient nutrients from the axenic medium, resulting in an undernourished organism [77]. Usually, glucose is added to axenic medium as the main energy source, though it can be replaced by other carbohydrates such as glycogen, fructose, and sucrose, but also by acetate [14].

For *D. melanogaster* , a so‐called holidic diet has been continuously refined over the last eight decades [10, 12, 78, 79, 80, 81]. An important milestone was the replacement of the protein source casein by individual amino acids [81], which enabled the variation of the amino acid composition and eventually led to the development of a holidic diet with an exome‐matched amino acid proportion [10]. For exome matching, an in silico translation of the *Drosophila* genome was carried out in order to estimate the relative distribution of the 20 proteinogenic amino acids in the proteome and, in the next step, to establish their ratios in the holidic fruit fly diet on this basis. Compared to all previous chemically defined media, the exome‐matched diet improved the fitness parameters fecundity and life expectancy in adult flies to the level of complex diets. However, this medium did not support optimal larval development of the fruit fly, which is likely related to the differences in metabolic physiology identified for these stages [82]. Recently, Sorge et al. [11] systematically optimized the nutrient composition of the holidic diet, resulting in the HolFast medium, in which the egg to adult development proceeds similarly to complex diets in terms of the three key traits mass gain, developmental speed, and survival. The crucial point was that an optimal ratio of amino acids in the diet was only effective if the vitamin and sterol composition and quantities were co‐optimized. Thus, similar to the situation in other animals, including humans [83, 84], the nutrient requirements of the fruit fly are stage‐specific and change throughout the lifecycle.

By definition, these chemically defined diets for *C. elegans* and *D. melanogaster* must, of course, contain all essential nutrients. Nevertheless, at least for *Drosophila*, two biofactors have been identified as essential, which have not been included in the previous holidic diet recipes. One is bromine, which is required for the assembly of the collagen IV scaffold of the basement membranes [85] (see below). Similarly, vitamin A has recently been shown to be essential for an intact visual system of the fruit fly [86] (see below). Similarly, molybdenum, which was recently discovered to be essential for *C. elegans* [87], is not yet a constituent of the nematode's chemically defined diet. Hence, future studies are warranted, where these biofactors will be added to the holidic diets. Of note, ascorbic acid (vitamin C), which is essential for humans but not for rodents, can be synthesized by both *C. elegans* and *D. melanogaster* and is therefore not a dietary requirement [88].

The following nutrients have been experimentally proven to be indispensable for both invertebrate organisms (see also Table 2):

- Sterols (cholesterol or ergosterol) [79, 91]
- Amino acids (arginine, histidine, lysine, tryptophan, phenylalanine, methionine, threonine, leucine, isoleucine, and valine) [12, 50, 81, 92]
- The water‐soluble vitamins thiamine (vitamin B1), riboflavin (vitamin B2), nicotinic acid (vitamin B3), pantothenic acid (vitamin B5), pyridoxine (vitamin B6), biotin (vitamin B7), and folic acid (vitamin B9) [45, 79, 93, 94, 95]

**TABLE 2:** The composition of chemically defined media for *C. elegans* and *D. melanogaster* .

Nutrient	CeMM *C. elegans*	Holidic diet *D. melanogaster*	Essential nutrients *M. musculus*
	[mg/L]	[mg/L]	[mg/kg]
Minerals			
Calcium	60^a^	68	5000
Copper	2.4^a^	1.0	6
Manganese	6.1^a^	0.28	10
Magnesium	72.5	50.5	500
Iron	11.6	5.0	35
Zinc	4.9	5.7	10
Iodine	X	X	0.15
Molybdenum	X^b^	X	0.15
Selenium	X	X	0.15
Salts			
NaHCO_3_ (Na)	X	1000	X
Sodium	n.d.^a^, ^c^	274	500
K_2_HPO_4_ (K, P)	1225.5	3000	X
K_3_‐Citrate H_2_O	486	X	X
Potassium	537	860	2000
Phosphorous	279	684	3000
Citric acid H_2_O	630.3	X	X
Acetic acid	X	3000	X
Amino acids (essential)			
L‐Arginine	975	1630	3000
L‐Histidine	283	650	2000
L‐Isoleucine	861	1120	4000
L‐Leucine	1439	2030	7000
L‐Lysine	1030	1370	4000
L‐Methionine	389	600	5000
L‐Phenylalanine	803	1010	7600
L‐Threonine	717	1110	4000
L‐Tryptophan	184	320	1000
L‐Valine	1020	1200	5000
Amino acid (non‐essential)			
L‐Alanine	1395	1100	X
L‐Aspartic acid	1620	1170	X
L‐Cysteine	19	340	X
L‐Glutamate	432	1520	X
L‐Glutamine	1463	1120	X
Glycine	722	770	X
L‐Proline	653	980	X
L‐Serine	788	1380	X
L‐Tyrosine	272	930	X
Carbohydrates			
D‐Glucose	32,500	X	X^b^
Sucrose	X	17,120	X^b^
Nucleotides			
Adenosine‐3′(2′)‐phosphoric acid	365.2	X	X
Cytidine‐3′(2′)‐phosphoric acid	323.2	X	X
Guanosine‐3′(2′) PO_4_ Na_2_ *H_2_O	363.2	X	X
Uridine‐3′(2′) phosphoric acid	324.2	X	X
Thymine	126.1	X	X
Inosine	X	65	X
Uridine	X	60	X
Water soluble vitamins			
Pantotheonate (Ca^2+^)	11.25^e^	10.9	16.0
Pyridoxine *HCl	15.0^f^	1.7	8.0
Riboflavin‐5′‐PO_4_ (Na^+^)	7.5	0.7	7.0
Thiamin *HCl	7.5	1.4	5.0
Biotin	3.75	0.15	0.2
Niacin	15.0^g^	8.4	15.0
Folic acid	7.5^h^	0.5	0.5
Cobalamin (vitamin B_12_)	3.75	X^i^	0.010
Choline	31.2	37.3	823
N‐acetylglucosamine	15	X	X
Pteroylglutamic acid	7.5	X	X
DL‐Thioctic acid	3.75	X	X
Glutathione (reduced)	204	X	X
myo‐Inositol	64.5	5	X
Fat soluble vitamins			
Retinol (A)	X	X^j^	0.72
Cholecalciferol (D)	X	X	0.025
*RRR*‐α‐tocopherol (E)	X	X	22.0
Phylloquinone (K)	X	X	1.0
Sterols			
β‐Sitosterol/	50	X^k^	X
Cholesterol	X^k^	300	X
Additional growth factor			
Haem (cytochrome c)	50	X	X

*Note:* The ingredients of the chemically defined CeMM [14] and the holidic exome‐matched diet [10] are listed. Nutrients that have proven to be essential for the respective species are marked by the blue‐shaded cells. Nutrients that are very likely required but have not yet been analyzed for essentiality are in gray shaded cells. *C. elegans* depend on a carbohydrate source such as glucose or, alternatively, a short chain fatty acid such as acetate. For *D. melanogaster* , the medium must contain a carbohydrate source such as glucose or sucrose. For comparison reasons, essential nutrients for mice are listed [84].

^a^ Essentiality is likely, however, complete deficiency of these minerals in the CeMM medium was difficult to induce [89].

^b^ Molybdenum was recently demonstrated to be essential for *C. elegans* [87].

^c^ The amount of sodium in the CeMM medium was not determined (n.d.) [89].

^d^ Mice diets usually contain high carbohydrate contents, although growth is also supported by a carbohydrate‐free diet [90].

^e^ 3.75 mg Pantethine + 7.5 mg Pantotheonate (Ca^2+^).

^f^ 3.75 mg Pyridoxamine *2HCl + 3.75 mg Pyridoxal PO_4_ + 7.5 mg Pyridoxine *HCl.

^g^ 7.5 mg Niacinamide + 7.5 mg Niacin.

^h^ 7.5 mg p‐Aminobenzoic acid.

^i^ Vitamin B_12_ is not added to the *Drosophila* holidic diet, however, may be provided by the microbiota.

^j^ Essentiality for the visual system has been recently demonstrated [86], however, not considered in holidic diet recipes so far.

^k^ Both sterols promote the growth of either *C. elegans* or *D. melanogaster* .

The metal ions of sodium, potassium, iron, calcium, magnesium, zinc, copper, and manganese play such a fundamental role in biological systems that they are considered to be essential here, even though their requirements have not all been tested in *D. melanogaster* and/or *C. elegans* .

- Sodium (Na) [12, 95], potassium (K) [95], calcium (Ca) [79], iron (Fe) [96, 97], magnesium (Mg) [95] and phosphorous (P) [79, 89, 95], zinc (Zn) [98, 99], copper (Cu) [100], manganese (Mn) [101]

Nutrients that have been demonstrated to be required by *D. melanogaster* but not (or not yet shown) by *C. elegans* are:

- Choline [79]
- Bromine [85]
- Retinol (vitamin A) [86]

Nutrients shown to be required by *C. elegans* but not (or not yet shown) by the fruit fly are:

- Haem [96]
- Cobalamin (B12) [102]
- Molybdenum [87]

Although molybdenum was shown to be required for larval development of *C. elegans* [87], it is not included in the chemically defined recipes so far. The fruit fly contains enzymes for the synthesis of the molybdenum cofactor (Moco), which is used by several molybdoenzymes [103]. However, the essentiality of molybdenum has not yet been demonstrated for the fruit fly. For cobalamin, as well, the question of its essentiality for the fruit fly remains unresolved [104].

Compared to the mammalian model organism mouse [84], current knowledge indicates that neither invertebrate requires:

- The fat‐soluble vitamins cholecalciferol (D), RRR‐α‐tocopherol (E), phylloquinone (K)
- Iodine
- Selenium.

Although *D. melanogaster* expresses three selenoproteins, selenium is non‐essential for the fruit fly [105]. Similarly, dietary selenium is not required in *C. elegans* , where thioredoxin reductase‐1 (TRXR‐1) is the sole selenoprotein [106].

Interestingly, a genetic study on gustatory receptors [107] revealed a stage‐specific requirement for RNA or RNA precursors such as uridine and inosine for *D. melanogaster* larvae. These molecules were found to be needed to achieve rapid development and optimal viability and are included in the HolFast diet formula accordingly [11].

### 3.6 Strengths and Limitations of *C. elegans* and *D. melanogaster* as Nutritional Models

Both invertebrate model organisms, *C. elegans* and *D. melanogaster* , offer a versatile methodological toolkit that has been increasingly employed in recent years to address a broad spectrum of questions in nutritional science [4, 5]. Three areas of research in which the advantages of the two organisms overlap are worth mentioning as examples. (i) Owing to their short generation times, the two models have been intensively used to study the bioactivity of nutrient factors with respect to lifespan and healthspan [108, 109]. (ii) *C. elegans* and *D. melanogaster* are also suitable to investigate the impact of biofactors, malnutrition, food deprivation and other dietary interventions on locomotion and foraging behavior [110, 111, 112, 113]. (iii) Both invertebrate models are applicable for high‐throughput tests due to their small body size. Accordingly, they are increasingly being used as in vivo models in food toxicology studies and similar large‐scale approaches [114]. As they are both genetic models in the first place, not only wildtypes, but also mutants and transgenics have been successfully applied in all these areas to decipher underlying metabolic and signaling pathways or to investigate the influence of nutritional factors on disease models [4, 5, 8]. However, one has to keep in mind that such disease models are often unable to cover all aspects of the complex and sometimes multifactorial nature of human diseases.

Due to their evolutionary distance from humans, the use of *C. elegans* and *D. melanogaster* presents additional fundamental nutrition‐related challenges that apply to both model organisms. For instance, mammals are endothermic and regulate their body temperature to maintain metabolic processes, whereas both invertebrates are ectothermic. This difference primarily affects energy expenditure and metabolic rate. Furthermore, the life cycles of *C. elegans* and *D. melanogaster* differ considerably from that of mammals. In the fruit fly, it includes a metamorphosis step that does not exist in mammals. Under unfavorable nutritional conditions, the life cycle of *C. elegans* can encompass a dauer larvae for which there is also no equivalent in humans. What fruit flies and mammals have in common is that reproduction primarily takes place at an early adult stage. Although the fruit fly has a drastically shorter life expectancy than laboratory rodents or humans, the kinetics of the survival curves are very similar in all cases. Nevertheless, it is still unclear to what extent this clear difference in life expectancy (months versus years) is associated with distinct aging mechanisms and/or hampers the transferability of results from, for example, life‐span studies with long‐term intervention. This applies even more so to *C. elegans* , which has an even shorter life expectancy.

In addition, both invertebrate models may as well differ from humans in their pharmacokinetic properties, which can, for example, affect the bioavailability of biofactors, with substantial consequences for the translation of dosage and treatment regimens. Traditional dose extrapolation between mammalian models (e.g., rodents) and humans relies on allometric scaling, assuming that physiological variables such as clearance, metabolism, and lifespan scale predictably with body mass [115]. Comparable calculations cannot readily be applied to invertebrate models because (i) the absorption and distribution of bioactives, (ii) metabolism, (iii) body temperature regulation, (iv) lifespan, and (v) the microbiome differ considerably between invertebrates and mammals/humans. Consequently, an equivalent of the FDA‐style “human equivalent dose” does not exist for these models. In some cases, determining the internal concentration of a compound required to elicit bioactivity in the invertebrate model may provide an indication of the effective dose range to be tested in mammals. Overall, however, studies using *C. elegans* and *D. melanogaster* are primarily intended to provide proof of principle regarding efficacy and underlying molecular mechanisms, rather than evidence for a quantitatively translatable human dose.

Despite these shared strengths and limitations, the two models differ considerably in some respects in terms of their suitability for nutritional research (Boxes 2 and 3), as explained below.

BOX 2 **Advantages of the *C. elegans* model in nutritional research compared to *D. melanogaster* .**

- Body transparency enables direct microscopic inspection/visualization of organs and subcellular organelles such as lipid droplets using dyes or marker proteins.
- Whole‐body respirometry is relatively simple.
- Shorter life cycle enables rapid nutritional intervention cycles.
- Self‐fertilization facilitates genetics, for example, nutrigenetic screens
- Genome‐wide gene silencing via RNAi (e.g., via RNAi feeding) is easier and more efficient
- True high‐throughput methodology including automation can be applied.

BOX 3 **Advantages of the *D. melanogaster* model in nutritional research compared to *C. elegans* .**

- Dietary flexibility is higher
- Only one defined (oral) uptake route of biofactors from food
- Quantitative and qualitative determinations of feed intake and excretion are possible
- Sex‐specific studies are possible
- The degree of genetic similarity to humans is higher.
- Innate immune system is more complex at the cellular and molecular level
- The anatomy is more complex
- Tissue‐specific analyses are possible
- Cell culture enables mechanistic studies.

#### 3.6.1 Handling, Morphology and Anatomy

*C. elegans* offers several practical advantages, including lower maintenance costs, ease of handling and long‐time storage which are clear benefits of the nematode over the fruit fly system. The transparent body of *C. elegans* allows for the microscopic examination of the inner cellular and subcellular anatomy [6]. Consequently, the impact of a dietary intervention on the spatial and temporal expression of GFP‐marker protein or the staining of structures such as lipid droplets can be monitored relatively easily in real time over the entire life cycle of the worm without further preparation, something that is hardly possible in the fruit fly, especially in the adult stage due to its melanized exoskeleton. Bioenergetic measurements are a crucial aspect when studying the influence of dietary regimes or biofactors on energy metabolism. Owing to its small body size, whole‐body respirometry can be performed on *C. elegans* [116], however, whilst this is relatively straightforward, it does not allow for organ‐specific resolution. In contrast, tissue isolation or mitochondrial purification is required for *D. melanogaster* , which is considerably more labor‐intensive but yields tissue‐specific results. The shorter life cycle of *C. elegans* compared to the fruit fly enables faster nutritional intervention cycles, which is advantageous, for example, in transgenerational nutritional studies. Another point in favor of its handling is the self‐fertilization of *C. elegans* , which facilitates genetic screening in general, and genetic nutritional screening in particular [6]. While some successful steps have been taken to integrate *Drosophila* into high‐throughput screening (HTS) systems, *C. elegans* has an advantage in this respect, being naturally HTS‐compatible. This advantage is particularly true of the possibilities for true automation, which are limited in *Drosophila* and therefore still require considerable manual work. In addition, RNAi is highly compatible, effective, and easy to implement [117, 118]. This allows nutritional and genetic studies to be combined helpfully in an HTS format, which is much more challenging to achieve with *Drosophila*.

The structural simplicity of the *C. elegans* model has both advantages and decisive disadvantages. Of particular note here is the organ composition, which is incomparable to that of humans and utterly different from that of *Drosophila*. Also linked to this are the phenomena associated with eutely, which have no counterpart in humans and *Drosophila*. The simplicity of the model prevents complex readouts, as the behavioral repertoire of *C. elegans* is very limited. In addition to this obvious methodological issue, others must be considered. These include the inability to manipulate the microbiota, particularly in the context of HTS studies, and the significant risk of contaminating the cultures with various microorganisms, each of which can drastically affect food manipulation and the integrity of the research.

Although the anatomy of *D. melanogaster* is simpler than that of vertebrates, it is more complex than that of *C. elegans* . As mentioned above, the nematode lacks many organs found in higher evolved organisms, which can limit studies of nutrient interactions that involve these complex systems. Two examples of food‐induced tissue‐specific pathologies that can only be studied in the *Drosophila* but not in the nematode are the development of renal stones [119] and heart dysfunctions [120] in fruit flies fed a high‐sugar diet. Nevertheless, certain human organs such as a closed circulatory system, lungs, or a sophisticated immune system often have only analogous counterparts in the fly or a corresponding organ is missing completely. The much simpler nervous system of fruit flies compared to mammals may limit their usefulness in investigating higher cognitive functions or brain disorders. In addition, the significantly smaller body size of *C. elegans* than that of the fruit fly makes working with tissues much more difficult or even impossible in the worm. In *D. melanogaster* , on the other hand, it is comparatively easy to dissect organs such as the intestine, the Malpighian vessels, the fat body, or the brain and use them for organ‐specific gene expression analyses, for example.

#### 3.6.2 Nutrient Requirements and Dietary Flexibility

The special dietary situation of *C. elegans* , characterized by marked differences in nutritional requirements, partly restricts the effectiveness in nutritional studies [121]. These requirements also differ significantly from our own in terms of quality. Furthermore, it is challenging to translate desired changes in food composition into an adequate diet for *C. elegans* , and this approach is not always successful [122, 123]. Related to this are the limited variability of food intake and the lack of a means to monitor food consumption (see below). Another issue in nutritional studies on biofactors that has to be considered when using standard *C. elegans* media with *E. coli* as the main food source is that positive or negative influences of nutritional factors on food bacteria can represent a serious confounding factor. In addition, biofactors may be metabolized by the bacteria, which can also significantly compromise the validity of the feeding experiments.

Chemically defined media exist for both model organisms. However, the composition of *C. elegans* media such as the CeMM has not been optimized as systematically on the basis of in silico and analytical data as the holidic diets for *D. melanogaster* [10, 11, 124]. Moreover, owing to their particle feeder nature, worms maintained under these axenic conditions usually exhibited a caloric restriction phenotype, as indicated by slower growth, increased lifespan, and metabolic changes [5]. Recently, an axenic, liposome‐based nanoparticle culture medium was developed to overcome these limitations to a large extent [77], yet it has rarely been used. Up to now, the CeMM medium was not appropriate to decipher the essentiality of minerals such as calcium, copper, manganese, and sodium [89]. In contrast, the holidic diets designed for *D. melanogaster* support development [11, 124] as well as adult fecundity and longevity in a manner similar to that of the complex media [10], enabling controlled studies on, for example, bioactivity, nutrient requirements and gene–nutrient interactions [10, 12, 124, 125, 126, 127].

Closely linked to this is the generally greater dietary flexibility of *D. melanogaster* , which is a major advantage for the use of the fruit fly as a nutritional model. As mentioned above, the composition of the standard complex diets in fruit flies can be modified relatively easily in terms of macronutrient ratios. This was successfully applied in the aforementioned studies on diet geometry [68]. Comparable studies have not yet been carried out in *C. elegans* , mainly because monoxenic culturing on complex diets with *E. coli* as food is not suitable for this purpose. The food bacteria are rich in protein with a proportion of about 55% and very poor in fat (7%–9%) and carbohydrates (6%) [45], making them very different from human diets. Moreover, while high‐sugar diets are established for both species [73, 128], high‐fat diets containing lard or coconut oil, for example, can only be studied in the *Drosophila* model, at least so far [75, 129, 130].

Although there is a remarkable overlap between the fruit fly and mammals in terms of those nutrient factors that have been identified to be essential (see Table 2), the nutritional requirements for *D. melanogaster* have not been conclusively clarified despite the development of holidic diets. Some nutrients, for example, that have been reported to be essential for the fruit fly, but also for mammals including humans, such as bromine or vitamin A, have not yet been included in these diets. Thus, their role must be further analyzed in this context. However, it is considered certain that *D. melanogaster* does not require the remaining fat‐soluble vitamins D, E, and K as well as the trace elements iodine and selenium. Accordingly, these vitamins and trace elements cannot be studied in the fly model to mimic human conditions. Finally, the role of vitamin B12 and molybdenum, which are currently not considered necessary, has not yet been conclusively clarified in terms of their essentiality.

Several aspects of human nutrition have been found to show sex‐specific differences [131, 132]. This also holds true for data from animal models [132], as males and females often exhibit different nutritional needs and differences in nutrient digestion and utilization. Studies on the role of sex dimorphism in nutrition are possible with the fruit fly, in which males and females occur in equal proportions under standard conditions, whereas the hermaphrodite *C. elegans* , in which males occur only sporadically, is not suitable for this purpose. Accordingly, sex‐specific effects of dietary regimens or biofactors have been frequently reported in nutritional studies with *D. melanogaster* [64, 132, 133, 134, 135].

#### 3.6.3 Food Intake, Digestion and Transport of Food

A central aspect of many nutritional intervention studies is the estimation of feed intake. However, quantification of feeding behavior and nutrient intake is more advanced in *D. melanogaster* than in *C. elegans* . Various methods have been developed for this purpose in *D. melanogaster* [136], with the recently introduced “Excretion quantification assay” being a simple, powerful, and quick test that can, in principle, be carried out with any solid *Drosophila* medium [137]. Additionally, this test can simultaneously measure the amount of ingested food remaining in the intestine and the amount excreted. This means that not only can the amount of food ingested be quantified, but also the excretions can be collected for further quantitative and qualitative analyses of their composition [138]. Feed intake assays have also been described for *C. elegans* ; however, these are significantly more complex, as they require specific technical equipment and can only be used to determine the pharyngeal pumping rate or the intake of feed bacteria [139, 140, 141]. These methods can therefore be used to determine relative changes in feed intake, but not to quantify the amount of diet that has been consumed. Hence, when supplements are added to solid NGM or liquid *C. elegans* media, it is difficult to assess how much of the supplement is taken up by the worms [5]. This is further complicated by the fact that the entire body of the worm is exposed to the diet, and it is often not clear whether supplements are ingested via the pharynx, which represents food intake in a strict sense, or via the cuticle or exposed neuronal endings [5]. Compared to this, the uptake of food and supplements in adult *D. melanogaster* occurs exclusively through the oral route and is therefore better defined. In addition, no method has been developed to collect or quantify the excretion of *C. elegans* .

Although the digestion of proteins and carbohydrates appears to be quite similar between humans and fruit flies, one limitation of *Drosophila* as a model for nutrition research is its distinct lipid digestion. It includes lipases but does not include bile acids, which are crucial for the intestinal fat metabolism in mammals [70, 71]. Another constraint of *D. melanogaster* as a nutrition model is its partially distinct mechanisms of nutrient absorption. For example, the fruit fly lacks an intestinal glucose transporter homolog to the mammalian sodium‐dependent glucose transporter 1 (SGLT1). Hence, in contrast to the enzymes α‐amylase and α‐glucosidase involved in poly‐ and disaccharide breakdown, the intestinal glucose transport of the fruit fly can only be used as a model for pharmacological studies to a very limited extent, if at all. In addition, most of the intestinal transport systems for absorbing amino acids and lipids are not yet well characterized in *D. melanogaster* . It remains to be seen whether the recently generated *Drosophila* intestinal cell line [57], which was reported to be capable of forming polar epithelial‐like structures in vitro, can be used to achieve breakthroughs in the field of intestinal nutrient transport.

Unlike in mammals, where glucose is the main transport form of sugar in the blood, the disaccharide trehalose takes over this task in the haemolymph of *Drosophila*. In contrast to glucose, trehalose is an inert molecule which does not contribute to nonspecific glycation of proteins, a deleterious modification that usually occurs in diabetic mammals. It is discussed that the trehalose content in the fruit fly is not subject to homeostasis for this reason and that the *Drosophila* insulin analogues primarily control the sugar uptake in muscles and the fat body as well as the formation of the energy stores glycogen and TAG [142].

#### 3.6.4 Metabolic and Signaling Pathways

At the molecular level, both *C. elegans* and *D. melanogaster* share many conserved metabolic and signaling pathways with humans (Table S1). This includes insulin/insulin‐like growth factor (IGF) signal transduction via the insulin receptor/ *C. elegans* DAF‐2, the pathways of AMP‐activated protein kinase (AMPK) and mitogen‐activated protein kinases (MAPK) as well as the pathway of the transcription factor nuclear factor erythroid 2‐related factor 2/ *C. elegans* skinhead‐1 (Nrf2/*Ce*SKN‐1) and histone deacetylases of the sirtuin family [5, 71]. Accordingly, both invertebrate models have significantly contributed to our knowledge on the biochemistry, molecular biology, and physiology of nutrition. As far as the physiology is concerned, there are some differences in hormonal regulation and metabolic equipment. In the fly, for example, in contrast to the mammalian system, glutamate rather than acetylcholine is released as a transmitter at the neuromuscular junction [143].

#### 3.6.5 Immune System and Infection

The genome of *D. melanogaster* generally shows greater homology to the human genome than that of the nematode, meaning that, in certain respects, only the fruit fly is suitable as a model organism. This applies, for example, to the molecular architecture of the innate immune system (Table S1). In fruit flies, as in humans, pathogens are recognized by canonical Toll‐like receptor signaling, which leads to an immune response via stimulation of the nuclear factor kappa‐light‐chain‐enhancer of activated B cells (NF‐κB) homolog Relish [144]. In contrast, *C. elegans* lacks both elements. A further difference can be found in the immune‐related Janus kinase (JAK)/signal transducer and activator of transcription (STAT) signaling pathway, which is activated in fruit flies and mammals via cytokines or cytokine‐like proteins and regulates the expression of immune effectors [144]. Neither a JAK homolog nor the upstream cytokine‐like proteins are present in the nematode. These differences in the molecular setup extend to cellular immunity. While the fruit fly possesses haemocytes that are present in the haemolymph and have a function similar to leukocytes in mammals [144], *C. elegans* also lacks a cellular immune defense.

Both *C. elegans* and *D. melanogaster* have been used as non‐mammalian infection models [145]. In the fruit fly, bacterial or fungal infections can be introduced in two ways: orally or systemically, via pinpricking or capillary injection. For the latter, protocols have been established that allow the application of a defined number of pathogens [146]. Moreover, in *D. melanogaster* , the bacterial load after oral infection can be determined by serial dilution of homogenates of infected flies and subsequent plating on appropriate agar plates [147]. In *C. elegans* studies, however, the oral route of infection is almost exclusively chosen because injection of bacterial or fungal solutions into the worm is extremely difficult. One drawback of this approach is that, when pathogens that have a harmful effect or even kill *C. elegans* are administered orally, feeding behavior is impaired, which makes it virtually impossible to standardize the number of infectious bacteria or fungi ingested [145]. Moreover, in the nematode, the molecular response to an oral infection can be analyzed only at the whole organismic level, whereas in the fruit fly the intestine or some other organs can be dissected for further tissue‐specific analyses. Consequently, the fruit fly offers a broader methodological framework for studying interactions between nutrition (including biofactors), immunity, and host–pathogen dynamics.

#### 3.6.6 Gut Microbiota

The gut microbiome is a critical factor in nutrition research, influencing metabolism, immunity, and overall health. In *C. elegans* studies, the worm is typically exposed to only one bacterial species, namely its food source *E. coli* OP50, meaning that the nematode cannot establish a genuinely diverse microbiota under laboratory conditions. In contrast, even laboratory strains of *D. melanogaster* harbor a microbiome, which usually comprises various bacteria and fungi species, although it is much simpler than that of mammals. In addition, as the gastrointestinal tract of the fruit fly is too short to generate anaerobic conditions, the bacteria in the fruit fly gut presumably exist mainly under aerobic conditions. In contrast, most parts of the human intestine allow only anaerobic processes. The limited diversity and mainly aerobic nature of gut bacteria in *D. melanogaster* , on the one hand, simplifies analysis, but on the other hand, restricts its ability to model complex host–microbe interactions found in mammals. Furthermore, the fruit fly lacks an adaptive immune system, which plays an important role in mammalian responses to dietary changes and gut microbiota fluctuations. Due to these differences, findings from *Drosophila* studies regarding diet‐microbiome interactions may not fully translate to humans or other mammals. This has to be kept in mind particularly when studying dietary interventions targeting gut health, such as probiotics or prebiotics.

#### 3.6.7 Cell Culture

The availability of cell culture systems further differentiates the two invertebrate models. While no cell culture exists for *C. elegans* , which may be due to its strictly deterministic development and eutely, a range of cell lines are established for *D. melanogaster* . Cell culture studies can be valuable for uncovering molecular mechanisms. For instance, in an RNAi screening study aimed at investigating the molecular mechanism of zinc detoxification, the *Drosophila* S2R+ cell line was successfully employed to discover candidate genes that have been suggested as potential modulators of zinc homeostasis [148]. Remarkably, a subset of these genes later showed up in a *D. melanogaster* study that mapped quantitative trait loci (QTL) associated with zinc toxicity and zinc homeostasis [149]. Specifically, cross‐validation of the roles of the genes *GluRIB* and *Nup98‐96* in zinc resistance was established. Of note, one of the recently established *Drosophila* intestinal cell cultures was reported to form three dimensional structures that displayed epithelial polarity [57]. This might enable modeling gut functions in vitro including the conduct of transport and barrier studies. Similar to the introduction of holidic diets for the fruit fly animals, attempts have also been made to develop a chemically defined medium for *Drosophila* cell cultures [150].

### 3.7 Exemplary Nutritional Phenotyping Studies on Biofactors Using *C. elegans*

The selected feeding studies summarized in Table 3 highlight the wide range of applications for *C. elegans* as a model organism to elucidate the role of various biofactors. A key selection criterion was that findings that are relevant to issues concerning nutrition are transferable to mammalian models or humans, as evidenced by corresponding studies (see also Table S1).

**TABLE 3:** Exemplary nutritional phenotyping studies on biofactors with *C. elegans* .

Biofactor	Bioactivity, function and mechanism	Essentiality	References
Trace element/metals			
Zinc	Regulates its own homeostasis via an evolutionary conserved low‐zinc‐activation (LZA) element	Yes	[151]
Lithium	High‐dose lithium improved olfactory memory and affected locomotor behavior	Uncertain	[152]
	High‐dose lithium impairs reproduction		[153]
Iron	Low and high concentration can increase stress tolerance and longevity	Yes	[154, 155]
	Elevated dietary iron activated the *C. elegans* p38‐mitogen‐activated protein kinase PMK‐1		[155]
Vitamines			
Vitamin B12	Increased dietary supply affects one‐carbon metabolism, thereby regulating RAS/MAPK‐dependent cellular processes	Yes	[156]
	Promotes longevity and healthy aging		[157]
Vitamin D	Promotes longevity and improves health span; triggers expression of genes involved in innate immune response and metabolism of xenobiotics	Yes	[158, 159]
	Mitigated β‐amyloid‐induced paralysis in a DAF‐16/forkhead box protein O‐dependent manner		[160]
Amino acids			
Glycine	Promotes lifespan extension	No	[161]
L‐leucine	Shortens reproductive span	Yes	[162]
Biogenic amines/amino acid derivatives			
Trimethylamine	Acts as a food attractant Modulates development, shortens lifespan, promotes neurodegeneration	No	[163]
Ergothioneine	Serves as a H_2_S precursor, thereby promoting longevity and improving healthspan via cytosolic glycerol phosphate dehydrogenase activation and subsequent elevation of NAD^+^ level	No	[164]
Fatty acids			
Long chain fatty acids (e.g., palmitic acid)	Initiate early postembryonic development via binding to nuclear hormone receptor NHR‐49 with simultaneous inactivation of the TORC1 pathway	No	[165]
Polyphenols			
3‐O‐methyl‐quercetin	Extends lifespan and healthspan by inducing nuclear translocation of DAF16/FoxO and increasing SKN1/Nrf2 expression Acts synergistic with quercetin and luteolin	No	[166]
*Carya cathayensis* polyphenols	Enhances stress resistance and promotes longevity in a DAF‐16 (FoxO)/DAF‐2 (insulin receptor)‐dependent manner	No	[167]
Triterpenoids			
Ganoderic acid A	Extends healthspan and lifespan	No	[168]
*Postbiotics*			
*Lactobacillus paracasei* postbiotics	Extends lifespan and mitigates muscle decline in aging worms	No	[169]
Plant/fungus extracts			
*Ganoderma lucidum* extract	Extends lifespan in a autophagy‐dependent manner inhibits the TOR pathway	No	[170]
*Citrus aurantium* L. var. *amara* Engl. extract	Exhibits anti‐obesity activity via an MDT‐15‐mediated mechanism	No	[171]

Abbreviations: FoxO, Forkhead box O transcription factor; MAPK, mitogen‐activated protein kinase; MDT‐15, Mediator 15; NHR‐49, nuclear hormone receptor 49; Nrf2, nuclear factor erythroid 2‐related factor 2; RAS, Rat sarcoma virus; Sir2, Sirtuin 2; SKN1, skinhead 1.

#### 3.7.1 Trace Elements/Minerals

The role of dietary metals, for example, has frequently been investigated in the nematode, particularly regarding their homeostasis, toxicity, and influence on aging. Zinc is an essential trace element for organisms but can be harmful in excess. To maintain proper levels despite fluctuating supply, a coordinated response from various proteins involved in zinc metabolism is necessary. By combining bioinformatics and genetic tools, a low zinc activation (LZA) element was discovered in *C. elegans* that, together with the trans‐acting factors ELT‐2 and MDT‐15, promotes transcription of target genes under zinc shortage [151]. Remarkably, the LZA element also functioned in human cells under zinc deficiency, revealing a conserved low‐zinc homeostasis mechanism.

The alkali metal lithium is well known for its pharmacological effect on the nervous system, where it stabilizes mood and provides neuroprotection. Although it is present in low concentrations in the diet of humans and other animals (< 100 μg/g or < 10 μM), its status in terms of essentiality is unclear. Administration of a supraphysiological lithium concentration of 15 mM was recently shown to improve olfactory memory in worms and affect their locomotor behavior by a mechanism that depends on a reduction in diacylglycerol levels [152]. This may have implications for understanding lithium's pharmacological mode of action in humans. On the other hand, a similar lithium concentration (10 mM) impaired reproduction of the nematode, manifested by reduced egg production [153].

Iron is suggested as a critical contributor to the aging process. Accordingly, dietary intervention studies revealed that both high and low dietary iron levels extend the lifespan of *C. elegans* [154]. The underlying mechanisms are suggested to be reduced ferroptosis, a form of iron‐dependent cell death and/or disrupted iron–sulfur cluster formation under low iron conditions or hormetic responses that activate stress‐related pathways. In line with the latter, Bhat et al. [155] demonstrated that the increased iron content in a stress‐resistant mutant of the food bacterium *E. coli* OP50, which was attributable to altered Fe‐S cluster biogenesis, also elicited an enhanced stress tolerance and, additionally, longevity in *C. elegans* . This occurred in a *C. elegans* PMK‐1/p38‐Mitogen Activated Protein Kinase (MAPK)‐dependent manner.

#### 3.7.2 Vitamins

Similar to humans, *C. elegans* requires dietary vitamin B12 (cobalamin), which is a fundamental cofactor in the one‐carbon metabolism formed by the methionine and folate cycles. A vitamin B12‐rich diet (addition of 64 nM) was shown to induce several RAS/MAPK‐dependent processes in *C. elegans* including vulval induction, germ cell apoptosis, and oocyte differentiation [156]. For this, tissue‐specific shifts in one‐carbon metabolites triggered by vitamin B12 administration were necessary. Corresponding studies in several human cell lines suggest that the mechanism by which the one‐carbon metabolism activates the RAS/MAPK pathway may be conserved in humans. Apart from this, supplementation of 100–400 nM vitamin B12 in *C. elegans* leads to an improved health status in aging nematodes including a prolonged lifespan and enhanced learning and memory abilities [157]. Interestingly, this study additionally reported that individuals in a human cohort of centenarians maintain relatively high vitamin B12 serum levels.

Vitamin D (cholecalciferol) when administered at 25–400 μM has a beneficial impact on age‐related protein aggregation in *C. elegans* , and hence promotes longevity by a mechanism that requires the evolutionarily conserved stress response pathway genes *skn‐1*, *ire‐1*, and *xbp‐1* [158, 159]. In good accordance, administration of 1 μM cholecalciferol was shown to mitigate β‐amyloid‐induced paralysis in a *C. elegans* Alzheimer's disease model in a DAF16/FoxO‐dependent manner [160]. This fits in with the fact that supplementation studies in patients with Alzheimer's disease suggest a protective effect of vitamin D. This includes a reduction in β‐amyloid markers and improvements in several cognitive areas [172].

#### 3.7.3 Amino Acids and Derivatives

In another feeding study with *C. elegans* , supplementation with the amino acid glycine (added to the medium at 5, 50, and 500 μM) promoted an extension of the nematodes' lifespan, which is in line with data from rodents [161]. As the lifespan extension was abolished in mutant worms lacking a functional methionine cycle, these data emphasize the abovementioned crucial role of one‐carbon metabolism in longevity.

Impaired branched‐chain amino acid metabolism, which causes an accumulation of leucine, isoleucine, and valine, is frequently linked to age‐related phenotypes. Supporting this, supplementing the diet of *C. elegans* with 5 mM L‐leucine resulted in a shortened reproduction phase [162]. It was suggested that the branched‐chain amino acids and their metabolism may play a similarly influential role in reproductive health in higher organisms.

The common food components choline, carnitine, and lecithin serve as precursors for the formation of the tertiary amine trimethylamine (TMA) by the gut microbiota. This bacterial metabolite has been identified as a nutrient attractant for *C. elegans* , which perceives TMA via the G‐protein coupled receptor tyramine receptor TYRA‐3. The presence of 2 mM TMA modulates development and accelerates aging and neurodegeneration in the nematode model [163]. Given that TMA is a risk biomarker for atherosclerosis and diabetes in humans, and that the human olfactory receptor trace amine‐associated receptor 5 (TAAR5) is activated by TMA in vitro, it has been speculated that evolutionarily conserved signaling pathways may be involved.

Ergothioneine is an amino acid‐derived secondary metabolite that is formed in mushrooms and certain bacteria. Although it has not been found to be an essential nutrient, ergothioneine has been suggested as a “longevity vitamin” because animals absorb the compound from the diet via the evolutionarily conserved transporter protein ergothioneine transporter (ETT); furthermore, it promotes healthy aging in animals and in models of age‐related diseases [173]. Although ergothioneine has antioxidant properties, its molecular mode of action remains elusive. A recent study in *C. elegans* revealed that ergothioneine is a substrate of the cystathionine γ‐lyase leading to the production of H_2_S [164]. Consistent with this, the administration of ergothioneine caused an increased persulfidation of proteins including cytosolic glycerol‐3‐phosphate dehydrogenase (cGPDH), which is part of the glycerophosphate shuttle. In accordance with this, ergothioneine treatment led to elevated NAD^+^ levels and increased respiration rates in mitochondria. Using a cystathionine γ‐lyase knockout worm strain, it was demonstrated that this pathway is responsible for the longevity phenotype following ergothioneine supplementation at 450 μM. Of note, experiments conducted on mice and murine cell lines as part of the same study support the findings that administration of ergothioneine improved mitochondrial function via this very mechanism.

#### 3.7.4 Fatty Acids and Lipids

Nutritional factors play a crucial role in initiating developmental stages. Long‐chain fatty acids (LCFA) such as palmitic acid that were tested in concentrations from 1 to 1000 μM have been identified as sufficient to drive early postembryonic development in *C. elegans* in a dose‐dependent manner reaching a maximum effect at 500 μM [165]. In this context, LCFA act as ligands for the conserved nuclear hormone receptor NHR49/PPARα. In addition, the LCFA‐mediated development requires an inactive TOR pathway. Notably, the human ortholog PPARα is activated by the same LCFA and by mTORC1 inhibition. Therefore, it has been suggested that this could be an evolutionarily conserved mechanism in early postembryonic animal development, as free LCFA are present in mammalian milk.

#### 3.7.5 Plant/Fungi Extracts and Phyto‐/Mycochemicals

In a recent *C. elegans* study, supplementing the diet with 600 μM 3‐O‐methylquercetin, a naturally occurring flavonoid, extended the lifespan and healthspan of worms [166]. These beneficial effects require the nuclear translocation of the forkhead box O transcription factor DAF‐16/FoxO and the upregulation of SKN‐1/Nrf2 expression, both of which are evolutionarily conserved and can be linked to an underlying hormetic mechanism whereby exposure to mild stressors enhances resilience and overall health. Interestingly, 3‐O‐methylquercetin was additionally tested in combination with two other polyphenols, quercetin and luteolin, which revealed synergistic effects.

Wang et al. [167] evaluated a fractionated polyphenol extract from *Carya cathayensis* (hickory nut), rich in corilagin, sanguisorbic acid dilactone, catechin, and gallic acid 3‐O‐(6‐galloylglucoside), using the *C. elegans* feeding model. The polyphenol mixture, when administered at concentrations from 10 to 200 μg/mL, significantly increased the lifespan of the nematode in a dose‐dependent manner without adversely affecting its reproductive fitness. Moreover, treated aging worms exhibited improved locomotor activity and reduced lipofuscin accumulation, an established marker for aging. In line with this, similar health promoting properties of polyphenols from *C. cathayensis* (dose range 400–1200 mg/kg) have also been demonstrated in a mouse model [174].

The triterpenoid ganoderic acid A has been recently identified from a library of 805 natural products through a multistep screening process using several different cell lines and functional read outs as a compound exhibiting low toxicity, stable anti‐senescence effects, and promising pharmaceutical properties [168]. Its anti‐aging and stress‐protective effects were subsequently confirmed in vivo using the *C. elegans* model (doses administered: 10, 100, and 1000 μM). Following these results, ganoderic acid A was administered via different administration routes (oral gavage, dietary incorporation, and intraperitoneal injection) at a dose range from 15 to 120 mg/kg body weight to various murine aging models, where it demonstrated geroprotective effects in irradiation‐stimulated premature aging mice, natural aged mice, and western diet‐induced obese mice. This is therefore also a good example of how invertebrate models can be integrated into the discovery processes of pharmaceutically active compounds.

Postbiotics are defined as a preparation of inanimate microorganisms and/or their components that confers a health benefit on the host [4]. *Lactobacillus paracasei* strain D3‐5 is a probiotic of human origin. Feeding *C. elegans* dead *L. paracasei* D3‐5 cells (administered at a dose equivalent to 0.3 × 10^8^ colony forming units/mL), which are then classified as postbiotics by definition, extended the lifespan of the nematode and mitigated age‐related muscle loss [169]. In the same study, the *Lactobacillus paracasei* postbiotic provided in drinking water (the administered dose was an equivalent of 10^9^ colony forming units/mL) also exhibited health‐promoting effects in mice, preventing HFD‐induced metabolic dysfunctions, improving gut health, and enhancing physical and cognitive functions.

A wide variety of biofactors that may contribute to disease prevention, therapeutic effects, and overall human health are produced by plants, algae, or fungi. Extracts from these natural sources are often initially tested in model organisms to prove the presence of health‐promoting properties. An aqueous extract of the medicinal fungus *Ganoderma lucidum* (also known as Lingzhi or Reishi in traditional Asian medicine) was administered in doses of 2 and 20 mg/plate, which prolonged the lifespan of *C. elegans* in an autophagy‐dependent manner [170]. This lifespan extension was accompanied by inhibition of the TOR pathway and a reduction in the longevity marker Fibrillarin 1 (FIB‐1), which negatively correlates with the lifespan of the worm. Consistently, the fungal extract also induced autophagy and inhibited the mTOR pathway in human Huh7 cells when given at doses of 0.5%–2%. Similar results were obtained when a sub‐fraction of the extract, which consists of low‐molecular weight compounds (< 10 kDa), was tested. Notably, the aforementioned triterpenoid ganoderic acid A, which exhibits similar bioactivity, is one of the primary bioactive compounds of this fungus.

A flavonoid‐rich ethyl acetate extract derived from the blossoms of the citrus tree *Citrus aurantium* L. var. *amara* Engl. (bitter orange) (administered at a dose range from 0.25 to 1.00 mg/mL) exhibited anti‐obesity activity in *C. elegans* through a MDT‐15 (Mediator‐15)/MED15 (mediator complex subunit 15)‐dependent mechanism that modulated the expression of genes involved in lipid and glucose metabolism [171]. Consistently, this study demonstrated that the extract inhibited the differentiation of murine 3T3‐L1 preadipocytes into mature adipocytes and showed anti‐obesity activity in HFD‐fed mice (dose range 10–50 mg/kg/days).

### 3.8 Exemplary Nutritional Phenotyping Studies on Biofactors Using *D. melanogaster*

The enormous potential of the *Drosophila* model for experimental research on biofactors becomes evident when looking at the list of selected feeding studies in Table 4. Here too, as with *C. elegans* , studies were chosen that had been conducted in a similar manner in mammalian models or in humans (see also Table S1), and whose outcomes are pertinent to nutritional inquiries.

**TABLE 4:** Exemplary nutritional phenotyping studies on biofactors with *D. melanogaster* .

Biofactor	Bioactivity, function and mechanism	Essentiality	References
Trace elements/bulk elements			
Bromine	Is an obligate cofactor for peroxidasin catalyzing crosslinks of collagen IV scaffold of basement membranes	Yes	[85]
Lithium	Sub‐pharmacological level promotes longevity in a glycogen synthase kinase 3‐dependent manner and increases egg production	Uncertain	[175, 176]
Sodium	Elevated dietary concentrations trigger receptor endocytosis in peripheral sweet taste receptor neurons, likely as part of the body's sodium homeostasis	Yes	[177]
Vitamines			
Pantothenic acid (B5)	Modulates nociceptor‐specific heat nociception in an *acsl4* ^*−/−*^ pain model	Yes	[178]
	Rescues diverse defects of mutants deficient in the gene Transport and Golgi organization 2 (TANGO2)		[179]
Retinol (A)	Deficiency results in severe damages in the visual system, downregulation of visual pigments and phototransduction proteins, decreased expression of mitochondrial TCA cycle and respiratory chain proteins in the eye	Yes	[86]
Amino acids			
Methionine	Methionine restriction leads to prolonged lifespan and decreased egg production; dietary level affects lifespan and reproduction in a complex interplay with the actual supply of the other 19 amino acids	Yes	[180]
	The trade‐off between prolonged lifespan and decreased egg production under methionine restriction is abolished by folate supplementation		[181]
Biogenic amines			
Spermidine	Promotes longevity and healthy aging through induction of autophagy and enhancement of hypusination of eIF5A	No	[182]
	Reduces TAG stores in adult fruit flies in an Akh/glucagon‐dependent manner		[138]
Fatty acids			
n3‐PUFA	Alters the pattern of free fatty acids and glycerophospholipids, improves mitochondrial function and prolongs lifespan	Uncertain	[183]
	Plays a role in the development of the visual system		[184]
Edible fats and oils (with different fatty acid patterns)	The fatty acid profile of the fruit fly shifts according to the fatty acid profile of the fat source under HFD, the fat quality determines effect size for traits such as lifespan, climbing activity, or fertility response to HFD with different fat qualities reveals small overlap of 30 differentially expressed genes	Uncertain	[75]
Polyphenols			
EGCG	Reduces iron level, upregulates mitochondrial iron transporter mitoferrin, and prolongs lifespan	No	[185]
	Mitigates ferroptosis‐dependent defects in a Parkinson disease model		[186]
Curcumin	Improves impaired taste‐associated memory in an Alzheimer's disease model	No	[187]
	Reduces iron and cobalt stores in flies		[188]
Phytocannabinoids			
Cannabidiol	Attenuates pain perception	No	[189]
Postbiotics			
*Aspergillus oryzae* extract	Increases heat tolerance associated with downregulation of metallothionein genes	No	[190]
Plant/algae extracts			
*Saccorhiza polyschides* extract	Extends the lifespan of both male and female flies in a TOR‐dependent manner the effect is even more pronounced under HFD conditions	No	[191]
*Morus merozygia* root bark extract	Selectively inhibits maltase and caused reduction of TAG stores in a HSD‐induced obesity model of *D. melanogaster*	No	[74]

Abbreviations: Acsl4, acyl‐CoA synthetase long‐chain family member 4; EGCG, epigallocatechin gallate; eIF5A, eukaryotic translation initiation factor 5A; FoxO, Forkhead box O transcription factor; HFD, high‐fat diet; HSD, high‐sugar diet; PGC1α, peroxisome proliferator‐activated receptor γ co‐activator; PUFA, polyunsaturated fatty acids; TAG, triacylglycerides; TCA, tricarboxylic acid cycle; Tor, target of rapamycin.

#### 3.8.1 Trace Elements/Minerals

A particularly striking example is undoubtedly the *Drosophila* study, in which the essentiality of the trace element bromine was demonstrated for the first time [85]. Subsequent studies have confirmed that the peroxidasin‐catalyzed step is a universal mechanism in tissue development, making bromine an essential nutrient for mammals, including humans [192].

As already mentioned above, the alkali metal lithium possesses bioactive properties. Of note, even a sub‐pharmacological dose of dietary lithium of 1 mM prolonged the lifespan and also increased the egg production in fruit flies, which is often seen as a trade‐off [175, 176]. A supplementation study with mice using lithium doses in the range from 0.02 to 1.05 g/kg diet revealed that lithium promotes healthy aging in a mammalian animal; however, it did not exhibit a lifespan‐prolonging effect [193]. Moreover, in line with the positive effect on fruit fly fecundity, administration of 1 mM lithium was found to be able to stimulate the maturation of human primordial oocytes [194].

Sodium is an essential bulk nutrient involved in many fundamental cellular and bodily processes. However, as both an excess and a shortage of sodium have adverse consequences for mammals and *D. melanogaster* , dietary sodium intake must be regulated. This homeostasis is maintained, among other mechanisms, by the gustatory system and sweet taste receptors in particular. A recent study in fruit flies revealed that sodium homeostasis under high salt conditions (3 mM in control diet versus 13 mM NaCl in the high salt diet) is closely linked to peripheral sweet gustatory receptor neurons [177]. A direct exposure of these neurons to sodium was sufficient to induce desensitization in a cell‐autonomous manner mediated by receptor endocytosis. Remarkably, this adaptation showed a sex‐specificity, as in females sweet receptor internalization occurred by both clathrin‐ and C‐terminal binding protein‐dependent micropinocytosis, whereas in males solely the clathrin‐dependent pathway was activated by high sodium levels in the diet. In conclusion, this peripheral desensitization of sweet gustatory receptor neurons is suggested to function as an additional component in sodium homeostasis, helping to avoid excess salt intake in the fly.

#### 3.8.2 Vitamins

Dietary supplementation with 0.8 mg/mL pantothenic acid (vitamin B5) rescued the altered neuronal morphology and the defective heat nociception in the *D. melanogaster* acsl4 pain model [178]. The acyl‐CoA synthetase long‐chain family member 4 (Acsl4) converts long‐chain fatty acids to acyl‐CoA molecules, which is an essential step for their further use in fatty acid metabolism and cell signaling. In mammals, this catalytic step is involved in the conversion of arachidonic acid into eicosanoids, including prostaglandin E2 (PGE2), which triggers, among other functions, nociceptor hypersensitivity [195]. Pantothenic acid serves as a metabolic precursor for coenzyme A, and its deficiency has been interestingly linked to neuropathic pain in humans [196]. However, the exact mechanisms of how pantothenic acid affects pain perception remain to be explored. In addition, supplementation of 2 or 4 mM pantothenic acid rescued diverse defects in a *Drosophila* TANGO2‐deficiency mutant, which functions as a model for human TANGO2‐deficiency disease (TDD). Since vitamin B5 also restores a cellular trafficking process that was impaired in TANGO2‐deficient human fibroblasts, it was suggested to be beneficial for TDD patients [179].

As mentioned above, retinol (vitamin A) is currently not a standard ingredient of the holidic diets of *D. melanogaster* [10, 11]. However, recent studies on retinol deficiency using the fruit fly model indicated that, similar to the situation in humans [197], the vitamin is required for the development of a proper visual system in the eye of *Drosophila* [86]. It is therefore suggested that retinol should also be classified as an essential nutrient biofactor for the fruit fly.

#### 3.8.3 Amino Acids and Derivatives

Methionine is an essential amino acid for *D. melanogaster* and mammals including humans. On the other hand, a high methionine intake has been shown to be toxic, whereas methionine restriction can lead to a prolonged life expectancy [198]. Against this background, Lee et al. [180] used a defined diet in the *Drosophila* model to investigate the complex interplay between different methionine intakes at varying supplies of the other 19 amino acids and the effects on associated biological traits. A lifespan‐extending effect of methionine restriction (achieved by a reduction of methionine from 10 to 1 mM) was found solely under a general low amino acid status but not when the amino acid supply was high. Hence, the results point to a mechanism that is independent of general amino‐acid sensing by the Tor complex 1 (TORC1) complex. In a similar approach, Wei et al. [181] deciphered that the trade‐off between a prolonged lifespan and a decreased egg production under methionine restriction was abolished by an elevated folate (vitamin B9) supplementation (tested dose range 1.13–226.6 μM). Since both metabolites methionine and folate are involved in one‐carbon metabolism, it was suggested that this pathway could play a significant role here. Of note, this study illustrates that the fruit fly is also a suitable model for investigating interactions between different nutrients.

Adequate polyamine levels are required for crucial life processes such as cell proliferation and cell differentiation. The cellular polyamine pool consisting of putrescine, spermidine, and spermine is replenished by endogenous synthesis from amino acids via decarboxylation reactions, complemented by dietary supply. Recently, the supplementation of 2.5 mM spermidine, a dose that cannot be achieved through normal diet, was found to have a strong anti‐obesity effect on adult female *D. melanogaster* that depended on the actual protein content of the diet, with high protein level abolishing the effect [138]. Lowering the TAG through spermidine administration was associated with an elevated spermidine catabolism and required the Akh/Akhr pathway. In line with these data, anti‐obesity effects of spermidine administration (either derived from a specific spermidine‐producing microbiota or at a dose of 3 mM via the drinking water) were observed in feeding studies with rodents [199, 200]. Dietary spermidine has already attracted attention, since it extends lifespan in several model organisms, including *D. melanogaster* . In line with its anti‐aging property, supplementation with 3 mM spermidine was demonstrated to improve locomotor activity, cognition, memory, and mitochondrial function in aging flies [182], which was associated with an enhanced hypusination of the eukaryotic translation initiation factor 5A (eIF5A). The amino acid hypusine (Nε‐[4‐amino‐2‐hydroxybutyl]‐lysine), which is found exclusively in eIF5A, is generated by an evolutionary conserved posttranslational modification, in which an amino‐butyl group is transferred from spermidine to a specific lysine residue of the polypeptide chain. The degree of eIF5A hypusination was identified as a critical factor in the aging process.

#### 3.8.4 Fatty Acids and Lipids

Depending on the fat source used, high‐fat diets can considerably vary in their fatty acid composition. The impact of fat quality and quantity on the fruit fly was assessed by preparing HFD diets based on different edible oils and fats including butterfat, sunflower oil, olive oil, linseed oil, and fish oil [75]. When feeding 12% HFDs based on these different fat qualities, the impact on the fruit fly was fat‐quality‐specific in terms of the flies' fatty acid profiles as well as life history traits such as lifespan, climbing activity, or fertility. Moreover, only a small group of genes was similarly affected in their transcript level by these HFDs, indicating that the fatty acid profile plays an important role for the assessment of dietary fat sources.

A high dietary n3/n6‐PUFA ratio was found to improve mitochondrial function of fruit flies, which was associated with a prolonged lifespan [183]. This is in line with the beneficial effects of PUFA on human health [201]. Certain PUFAs cannot be synthesized in human metabolism and are therefore essential nutrients. It is currently still unclear whether *Drosophila*, which also lacks such a synthesis pathway, is essentially dependent on the supply of PUFAs from the diet. However, it has been shown that this class of fatty acids plays an important role in the development of the fruit fly's visual system, although a diet consisting predominantly of PUFAs had a negative effect on the rhabdomere structure [184].

#### 3.8.5 Plant/Fungi Extracts and Phyto‐/Mycochemicals

Green tea and, in particular, its polyphenol epigallocatechin gallate (EGCG) are attributed with health‐promoting effects [202]. This was confirmed by using the *D. melanogaster* model where the addition of 20 mM EGCG led to a prolongation of life and healthy aging. Lopez et al. [185] found that EGCG supplementation significantly reduced total body and mitochondrial iron levels most probably owing to its ability to bind free iron. This was accompanied by the upregulation of the mitochondrial iron transporter mitoferrin, which was demonstrated by mutant studies to be crucial for the longevity phenotype under EGCG supplementation. In line with these findings, a recent study using a *Drosophila* Parkinson disease model found that administration of 0.5 mM EGCG ameliorated several behavioral and neuronal defects in the mutant flies by restoring iron levels and thereby lowering ferroptosis [186].

Curcuminoids (collectively referred to as curcumin) are polyphenols with a diarylheptanoid structure that have long been known as remedies in traditional Asian medicine. Modern research has also examined the potential health benefits of curcumin in a wide array of studies, including its putative neuroprotective properties [203]. In a *D. melanogaster* Alzheimer's disease model, administration of 0.1% curcumin specifically restored the taste‐associated memory function of the mutant flies, while vitamin C, riboflavin, and metformin, which improved other impaired phenotypes in the Alzheimer's disease fly model, were ineffective in this case [187]. The physiological consequences of the in vitro proven metal‐chelating property of curcumin were examined by using the fruit fly model [188]. The addition of 0.2% curcumin led to an altered trace metal pattern in adult flies with reduced iron and cobalt levels. Remarkably, in previous feeding studies with mice, a diet containing 0.2% curcumin resulted in iron deficiency and splenomegaly [204, 205]. In summary, these data indicate that *D. melanogaster* is a suitable in vivo model to study trace metal biology.

Preclinical and clinical studies suggest that the non‐psychoactive phytocannabinoid cannabidiol is a promising candidate for pain management. In a recent study, cannabidiol was investigated in a *D. melanogaster* gain‐of‐function mutant of the voltage‐gated sodium channel Paralytic (*para* ^*bss1*^), which is a functional analogue of vertebrate Na_v_ channels and thus represents a fruit fly model for chronic nociception [189]. Consistent with its inactivation effect on human Na_v_1.7 channels, oral administration of 0.1 mg/mL cannabidiol promoted the attenuation of chemical nociception in *D. melanogaster* , confirming the analgesic properties of this phytocannabinoid.

A supplementation study carried out in *D. melanogaster* revealed that the addition of 5% of a postbiotic derived from the filamentous fungus *Aspergillus oryzae* to the fruit fly diet conferred an increased resilience against an acute heat stress [190]. Transcriptome analyses indicated that this was associated with downregulation of metallothionein B, C, and D genes involved in the modulation of oxidative stress and immunity. Remarkably, this study demonstrated that the same *A. oryzae* postbiotic product, when given twice daily for 26 days at a dose of 3, 6, or 18 g per cow per day, also induced heat tolerance in lactating dairy cows, probably via a mechanism that reduces inflammation.

Supplementation of the fruit fly diet with 0.1% of an aqueous extract of the marine brown alga *Saccorhiza polyschides* (furbelow) caused a significant extension of lifespan in both males and females [191]. This beneficial impact was even more pronounced under HFD conditions. The intestinal microbiota of *S. polyschides* extract‐treated flies exhibited increased species richness, and genetic analyses indicate that bioactive factors in the brown alga extract prolong lifespan by interacting with the TOR pathway. In a corresponding mouse study, supplementation with 5% of the *S. polyschides* extract significantly attenuated HFD‐induced increases in body weight and fat mass, while also counteracting adverse effects of long‐term HFD feeding [206]. Further analyses suggest a multifactorial underlying mechanism, including alterations in gut microbiota, likely driven by the fermentation of indigestible polysaccharides that constitute major components of brown algae.

The African medicinal plant *Morus mesozygia* is traditionally used to treat diabetes and hyperglycaemia. Evaluation of a *M. mesozygia* root bark powder (tested at the final concentrations of 1.0% and 2.5%) in a diet‐based *D. melanogaster* model for the in vivo pharmacological assessment of α‐glucosidase inhibitors revealed potent maltase‐inhibitory and carbohydrate‐ and TAG‐reducing activity without adverse effects on development or lifespan [74]. Consistently, a hypoglycaemic impact of an *M. mesozygia* extract was observed in a diabetes rat model [207]. In summary, these studies indicate that *M. mesozygia* contains biofactors with promising hypoglycaemic and anti‐diabetic activity.

## 4 Conclusion

**FIGURE 2:**
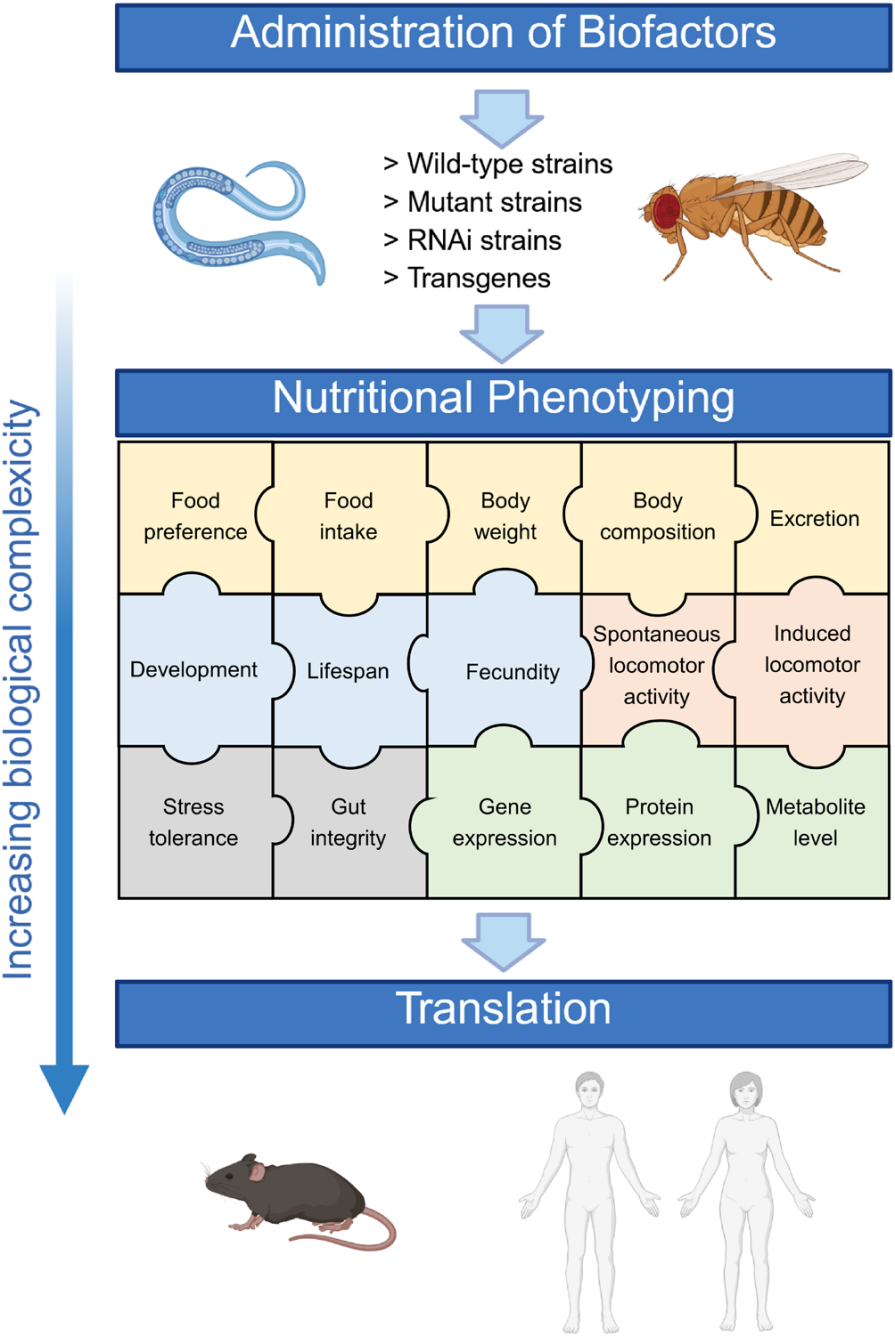
The experimental and conceptual use of invertebrate models in biofactor research. Biofactors are added to or omitted from the diets of the invertebrate models *C. elegans* or *D. melanogaster* . The impact of this intervention can be studied in wild‐type animals, mutants, RNAi strains, or transgenes by employing nutritional phenotypic analyses (examples of relevant phenotypes are depicted). Findings can subsequently be evaluated in biological models of greater complexity and ultimately in humans. Created in BioRender. Rimbach, G. (2026) https://BioRender.com/peipo0v.

*C. elegans* and *D. melanogaster* have proven to be highly versatile invertebrate models for investigating the physiological effects of biofactors, owing to their genetic tractability, short generation times, and cost‐efficient maintenance. Their utility is further enhanced by the availability of chemically defined diets. While *C. elegans* offers unique advantages for in situ microscopy, enabling real‐time observations of biological processes in living, intact animals, as well as for high‐throughput screening and genetic studies in a simple, well‐defined organism, it has limitations in terms of anatomical complexity. *D. melanogaster* presents a sophisticated platform, enabling organ‐ and tissue‐specific analyses, precise quantification of food intake, and modeling of complex, sex‐specific, and disease‐related phenotypes. Despite the evolutionary distance from humans, the fruit fly's higher degree of genetic and physiological homology—especially in metabolic, immune, and endocrine pathways—makes it an important model for many aspects of biofactor research. Taken together, the strategic use of these two complementary models can significantly advance our understanding of nutrient‐gene interactions and support the identification of novel biofactors with potential relevance for human health and chronic disease prevention (Figure 2). However, careful conceptual integration is required to translate findings obtained in *C. elegans* or *D. melanogaster* to more complex organisms and ultimately humans. A key consideration is the essentiality of the biofactor. Compounds that are universally required for cellular function, such as certain vitamins or trace elements, are more likely to exhibit conserved roles across species. Equally important is the conservation of metabolic pathways. If a biofactor participates in evolutionarily preserved biochemical processes such as mitochondrial energy metabolism or redox regulation, then findings from invertebrates can often be meaningfully extended to human biology. Conversely, limitations arise when pathways diverge or when organisms differ significantly in physiology. Bioavailability is another critical factor. Even if a biofactor exerts clear effects in an invertebrate model, differences in absorption, transport, and tissue distribution in humans may alter its efficacy. Additionally, dosage and exposure conditions in model organisms may not accurately reflect human nutritional contexts. Therefore, integrating findings requires a framework that considers both shared biological mechanisms and species‐specific differences. This ensures that conclusions drawn from invertebrate studies are applied to humans with appropriate caution and scientific rigor.

## Author Contributions

**Kai Lüersen:** writing – conceptualization, original draft preparation. **Thomas Roeder:** writing – original draft preparation. **Gerald Rimbach:** writing – original draft preparation. All authors have read and agreed to the published version of the manuscript.

## Funding

This work was supported by Deutsche Forschungsgemeinschaft, GRK3128, 544530435, CRC 1182 project C2.

## Conflicts of Interest

The authors declare no conflicts of interest.

## Supporting information


**Table S1:** Key genes from invertebrate models discussed in this review and their human orthologues. The genes are listed in the order of their appearance in the text. The orthologues were identified by using FlyBase, WormBase, and the cited literature.

## Data Availability

Data sharing not applicable to this article as no datasets were generated or analyzed during the current study.
