# Supplementary material for: Caenorhabditis elegans and Drosophila melanogaster as Model Organisms in Biofactor Research
Source: Biofactors. 2026 Jul 6;52(4):e70127. doi: 10.1002/biof.70127 (PMC13337977; doi:10.1002/biof.70127)
Supplement: Supplementary file 1 — Table S1: Key genes from invertebrate models discussed in this review and their human orthologues. The genes are listed in the order of their appearance in the text. The orthologues were identified by using FlyBase, WormBase, and the cited literature. [file BIOF-52-0-s001.docx]

**Table S1 Key genes from invertebrate models discussed in this review and their human orthologues.** The genes are listed in the order of their appearance in the text. The orthologues were identified by using FlyBase, WormBase, and the cited literature.

| ***C. elegans***  Gene (WormBase ID) | ***D. melanogaster***  Gene (FlyBase ID) | **Human**  Gene (Ensembl-ID) | **Encoded protein & function** |
| --- | --- | --- | --- |
| *daf-2* (WBGene00000898) | *InR* (FBgn0283499) | *IGF1R* (ENSG00000140443)  *INSR* (ENSG00000171105) | insulin/insulin-like growth factor receptor; master regulator of the conserved insulin/insulin-like growth factor signal transduction pathway |
| *aak-2* (WBGene00020142) | *AMPKα* (FBgn0023169) | *PRKAA1* (ENSG00000132356)  *PRKAA2* (ENSG00000162409) | AMP-activated protein kinase; master regulator of the AMPK pathway functioning as cellular energy sensor |
| Extracellular signal-regulated kinase (ERK) | |  | serine/threonine kinases; master regulators of the conserved mitogen-activated protein kinase pathway involved in proliferation, metabolism, stress response, immunity, and apoptosis |
| *mpk-1* (WBGene00003310)  *mpk-2* (WBGene00003311) | *rl* (FBgn0003256) | *MAPK1 (ERK2)* (ENSG00000100030)  *MAPK3 (ERK1)* (ENSG00000102882) |  |
| p38 mitogen-activated protein kinase | |  |  |
| *pmk-1* (WBGene00004055)  *pmk-2* (WBGene00004056)  *pmk-3* (WBGene00004057) | *p38a* (FBgn0015765)  *p38b* (FBgn0024846)  *p38c* (FBgn0267339) | *MAPK14 (p38 α)* (ENSG00000112062) |  |
| c-Jun N-terminal Kinases | |  |  |
| *jnk-1* (WBGene00002166)  *kgb-1*  (WBGene00002220) | *bsk (jnk)* (FBgn0000229) | *MAPK8 (JNK1)* (ENSG00000109339)  *MAPK9 (JNK2)* (ENSG00000165905)  *MAPK10 (JNK3)* (ENSG00000109332) |  |
| *skn-1* (WBGene00004804) | *cnc*  (FBgn0262975) | *NFE2L1* (ENSG00000082641)  *NFE2L2* (ENSG00000116044) *NFE2L3* (ENSG00000050344) | transcription factor nuclear factor erythroid 2-related factor 2; master regulator of the conserved Nrf2-pathway involved in cellular detoxification, oxidative stress responses, and metabolic homeostasis |
| *sir-2.1* (WBGene00004800)  *sir-2.2* (WBGene00004801)  *sir-2.3* (WBGene00004802)  *sir-2.4* (WBGene00004803) | *Sirt1* (FBgn0024291)  *Sirt2* (FBgn0038788)  *Sirt4*  (FBgn0029783)  *Sirt6*  FBgn0037802  *Sirt7* (FBgn0039631) | *SIRT1* (ENSG00000096717)  *SIRT2 (ENSG00000268579)*  *SIRT3* (ENSG00000142082)  *SIRT4  (ENSG00000089163)*  *SIRT5* (ENSG00000124523)  *SIRT6* (ENSG00000077463)  *SIRT7* (ENSG00000187531) | NAD^+^-dependent deacylases; master regulator of the conserved sirtuin pathway involved in cellular metabolism, DNA repair, epigenetics, and ageing processes |
| no orthologue | *Tl* (FBgn0262473)  *18w* (FBgn0287775)  *MstProx* (FBgn0015770)  *Toll-4* (FBgn0032095)  (five further receptors: *Tehao; Tol-6;  Toll-7; Toll-9; Tollo*) | *TLR1* (ENSG00000174125)  *TLR-2* (ENSG00000137462)  *TLR-3* (ENSG00000164342)  *TLR-4* (ENSG00000136869)  (six further receptors: *TLR5-TLR10*) | Toll-like receptors; involved in development and the innate immune response |
| no orthologue | *Relish* (FBgn0014018)  *dl* (FBgn0260632) | *NF-κB* (ENSG00000109320)  *NFKB2* (ENSG00000077150) | nuclear factor kappa-light-chain-enhancer of activated B-cells; transcription factor regulating the innate immune response |
| no orthologue | *hop* (FBgn0004864) | *JAK1* (ENSG00000162434)  *JAK2* (ENSG00000096968)  *JAK3* (ENSG00000105639)  *TYK2* (ENSG00000105397) | Janus kinases of the conserved JAK/STAT pathway; involved in the innate immune response, inflammation, growth, and development |
| no orthologue | *Stat92E* (FBgn0016917) | *STAT1* (ENSG00000115415)  *STAT2* (ENSG00000170581)  *STAT3* (ENSG00000168610)  *STAT4* (ENSG00000138378)  *(STAT5A* (ENSG00000171071)  *STAT5B* (ENSG00000173757)  *STAT6* (ENSG00000166888) | Signal-transducer and activator of transcription factor of the conserved JAK/STAT pathway; involved in the innate immune response, inflammation, growth, and development |
| *elt-2*  (WBGene00001250) | *srp*  (FBgn0003507) | *GATA4* (ENSG00000136574)  *GATA5* (ENSG00000130700)  *GATA6* (ENSG00000141448) | GATA-type transcription factor; involved in tissue development |
| *mdt-15* (WBGene00007016) | *med15*  (FBgn0027592) | *MED15* (ENSG00000099917) | mediator of RNA polymerase II transcription subunit 15; enables nuclear receptor binding activity and transcription coactivator activity; master regulator of lipid metabolism, and stress resistance |
| *let-60* (WBGene00002335) | *Ras85D* (FBgn0003205)  *Ras64A*  (FBgn0003206) | *KRAS* (ENSG00000133703)  *NRAS* (ENSG00000213281)  *HRAS*  (ENSG00000174775)  *RRAS* (ENSG00000133818)  *ERAS* (ENSG00000187682) | GTP-binding protein; key regulator of the RAS/MAPK pathway; plays key roles in multiple cellular and developmental processes |
| *ire-1*  (WBGene00002147) | *Ire1*  (FBgn0261984) | *ERN1* (ENSG00000178607)  *ERN2* (ENSG00000134398) | endoplasmic reticulum to nucleus signalling; serine/threonine-protein kinase and endoribonuclease; acts as a primary stress sensor in the unfolded protein response |
| *xbp-1* (WBGene00006959) | *Xbp1*  (FBgn0021872) | *XBP1* (ENSG00000100219) | X-box binding protein 1 transcription factor; serves as the master regulator of the endoplasmic reticulum (ER) unfolded protein response |
| *daf-16* (WBGene00000912) | *foxo*  (FBgn0038197) | *FOXO3* (ENSG00000118689)  *FOXO1* (ENSG00000150907)  *FOXO4* (ENSG00000184481)  *FOXO6* (ENSG00000204060) | forkhead box O transcription factor; serves as the master regulator of aging, stress resistance, and metabolic reprogramming |
| *tyra-3* (WBGene00006475) | *TyrR*  (FBgn0038542) | *TAAR5* (ENSG00000135569) | G-protein coupled receptor primarily for tyramine; major neuromodulator in stress responses, sensory perception, and behavioural plasticity |
| *cth-1*  (WBGene00009048) | *Cth* (FBgn0000566) | *CTH* (ENSG00000116761) | cystathionine γ-lyase; responsible for synthesizing L-cysteine and regulating trans-sulfuration pathways |
| *gpdh-1* (WBGene00009824) | *Gpdh1*  (FBgn0001128) | *GPD1* (ENSG00000167588) | cytolsolic glycerol-3-phosphate dehydrogenase; involved in NAD^+^ regeneration by the glycerol phosphate shuttle and lipogenesis |
| *nhr-49* (WBGene00003639) | *Hnf4*  (FBgn0004914) | *HNF4A*  (FBgn0003206)  *HNF4G* (ENSG00000164749)  *PPARα* (ENSG00000186951) | nuclear hormone receptor family member; RNA polymerase II cis-regulatory region sequence-specific DNA binding; key regulator of lipid metabolism |
| *let-363* (WBGene00002583) | *mTor*  (FBgn0021796) | *mTOR* (ENSG00000198793) | molecular target of rapamycin serine/threonine kinase in the TOR pathway; involved in nutrient and energy sensor; master regulator of translation, cellular metabolism, growth, and aging |
| *pxn-2* (WBGene00004257) | *Pxn*  (FBgn0011828) | *PXDN* (ENSG00000130508) | peroxidasin; bromine-dependent cross-linking of collagen IV molecules, providing structural scaffolding for basement membranes |
| *acs-4*  (WBGene00018152) | *Acsl*  (FBgn0263120) | *ACSL4* (ENSG00000068366) | acyl-CoA synthetase long-chain; regulates fatty acid metabolism |
| *hrg-9* (WBGene00011305)  *hrg10* (WBGene00013587) | *Tango2*  (FBgn0030503) | *TANGO2* (ENSG00000183597) | transport and Golgi organization 2; involved in cellular bioenergetics, lipid homeostasis, and oxidative stress homeostasis |
| *nlp-47* (WBGene00018117) | *Akh*  (FBgn0004552) | *GCG* (ENSG00000115263) | peptide hormone; regulates carbohydrate, lipid and glycogen homoestasis |
| *gnrr-1* (WBGene00018798) | *Akhr*  (FBgn0025595) | *GCGR* (ENSG00000215644) | G protein-coupled receptor; regulates carbohydrate, lipid and glycogen homoestasis |
| *iff-1*  (WBGene00002064)  *iff-2*  (WBGene00002065) | *eEF5*  (FBgn0285952) | *EIF5A* (ENSG00000132507) | eukaryotic translation initiation factor 5A; functions in translation elongation and termination |
| *mfn-1* (WBGene00012204) | *mfrn* (FBgn0039561) | *SLC25A37 (MFRN1)* (ENSG00000147454)  *SLC25A28 (MFRN2)* (ENSG0000015528) | mitoferrin; involved in mitochondrial iron transport and required for heme synthesis of hemoproteins and Fe-S cluster assembly |
| no orthologue | *para*  (FBgn0285944) | *SCN1A*  (ENSG00000144285)  *SCN2A*  (ENSG00000136531)  *SCN3A* (ENSG00000153253) | α-subunit of voltage-gated sodium channels; involved in neuronal excitability |
| *mtl-1*  (WBGene00003473)  *mtl-2*  (WBGene00003474) | *MtnB*  (FBgn0002869)  *MtnC*  (FBgn0038790)  *MtnD*  (FBgn0053192) | *MT1A* (ENSG00000205362)  *MT2A* (ENSG00000125148)  *MT3* (ENSG00000087250) | metallothioneins; involved in the homeostasis and/or detoxification of metal ions, as well as in the stress response |
| *aagr-3* (WBGene00009583)  *aagr-4*  (WBGene00018682) | *Mal-A1*  (FBgn0002570)  (eight further a-glucosidases: *MAL-A2-A8*, *MAL-B2*) | *MGAM*  (ENSG00000257335) | Maltase (a-glucosidase); involved in disaccharide digestion |
